# A Feel for Numbers: The Changing Role of Gesture in Manipulating the Mental Representation of an Abacus Among Children at Different Skill Levels

**DOI:** 10.3389/fpsyg.2018.01267

**Published:** 2018-08-07

**Authors:** Philip S. Cho, Wing Chee So

**Affiliations:** ^1^Underwood International College, Yonsei University, Songdo, South Korea; ^2^Institute of Convergence Science, Center for Science and Engineering Applications in Social Science, Yonsei University, Seoul, South Korea; ^3^Department of Educational Psychology, The Chinese University of Hong Kong, Shatin, Hong Kong

**Keywords:** mental arithmetic, abacus, gesture, mathematics learning, embodiment

## Abstract

Abacus mental arithmetic involves the skilled acquisition of a set of gestures representing mathematical algorithms to properly manipulate an imaginary abacus. The present study examined how the beneficial effect of abacus co-thought gestures varied at different skill and problem difficulty levels. We compared the mental arithmetic performance of 6- to 8-year-old beginning (*N* = 57), intermediate (*N* = 65), and advanced (*N* = 54) learners under three conditions: a physical abacus, hands-free (spontaneous gesture) mental arithmetic, and hands-restricted mental arithmetic. We adopted a mixed-subject design, with level of difficulty and skill level as the within-subject independent variables and condition as the between-subject independent variable. Our results showed a clear contrast in calculation performance and gesture accuracy among learners at different skill levels. Learners first mastered how to calculate using a physical abacus and later benefitted from using abacus gestures to aid mental arithmetic. Hand movement and gesture accuracy indicated that the beneficial effect of gestures may be related to motor learning. Beginners were proficient with a physical abacus, but performed poorly and had low gesture accuracy during mental arithmetic. Intermediates relied on gestures to do mental arithmetic and had accurate hand movements, but performed more poorly when restricted from gesturing. Advanced learners could perform mental arithmetic with accurate gestures and scored just as well without gesturing. These findings suggest that for intermediate and advanced learners, motor-spatial representation through abacus co-thought gestures may complement visual-spatial representation of a mental abacus to reduce working memory load.

## Introduction

Abacus arithmetic is an ideal model for examining the changing beneficial effect of co-thought gestures for learning mathematics at different skill and problem difficulty levels. According to Gesture as Simulated Action theory, learners spontaneously gesture to activate motor programs that assist working memory as imagery tasks pass a threshold of difficulty (Jeannerod, 2001; Hostetter and Alibali, 2008). Gestures, thus, are the “visible embodiment" of simulated actions that reveal implicit knowledge and strategies for solving mathematical problems and enhance learning (Goldin-Meadow et al., 2001; Broaders et al., 2007; Cook et al., 2010). We examine the beneficial effect of spontaneous co-thought gestures performed during mental arithmetic as the visible embodiment of mathematical algorithms for manipulating the mental representation of an abacus.

The role of co-thought gestures, i.e., non-communicative hand movements without accompanying speech, in mathematics learning is less well understood than co-speech gestures. In two recent papers, co-thought gestures were found to change from action simulation to representation of action plans (Chu and Kita, 2008, 2011, 2016; Alibali et al., 2011). Participants spontaneously produced more co-thought gestures the greater the angel of mental rotation and solved more problems correctly when allowed to gesture as opposed to the gesture-prohibited condition (Alibali et al., 2011; Chu and Kita, 2011). This beneficial effect of co-thought gestures was generalizable across tasks involving similar visual-spatial transformations. Co-thought gestures also changed over time with learning experience. Experienced participants not only gestured less than novices over subsequent trials solving spatial problems, but also changed the type of gestures they used. In a study of mental rotation, Chu and Kita (2008) found that learners’ spontaneous gestures changed from action simulation (e.g., curved palm rotates to the right so as to represent an act of rotating), to representation of an object with hands (e.g., flat palm flips outward so as to represent the object being rotated) in the subsequent trials. The authors proposed that both co-thought gestures and co-speech gestures are produced by the same action generation process, which internally represents and plans purposeful actions that have a direct physical impact on the world, such as manipulating an object or locomotion (Chu and Kita, 2016). To support their *action generation hypothesis*, they found that participants produced more co-thought and co-speech gestures when stimulus objects afforded actions (i.e., could be easily manipulated), compared to when objects were not easily handled (i.e., having spikes) (Chu and Kita, 2016). Similar to Chu and Kita’s motivation for proposing the action generation hypothesis, Pouw et al. (2014) have argued that there is a need to extend GSA to explain how co-thought gestures recruit the motor system to benefit cognitive processes.

No studies have examined the possible beneficial effect of co-thought gestures in learning mathematics. Early studies of abacus mental arithmetic have all noted that learners move their hands and fingers when performing mental calculations, as if manipulating a real abacus (Hatano and Osawa, 1983; Stigler, 1984). Yet, these studies have not explained why learners spontaneously gesture nor examined whether the beneficial effects of these hand gestures vary with the skill levels of learners and the difficulty levels of problems.

The abacus is an historically significant cultural artifact that affects how the brain processes calculations as part of a living tradition of embodied mathematics. Abacus arithmetic has become one of the most common and widespread forms of early childhood mathematics education throughout Asia. In the highly competitive educational systems of countries like Singapore, Taiwan, and South Korea, nearly all children attend supplemental classes till late in the evening. The trend has been for children to start at an increasingly early age, with most beginning around 5 or 6 years old.

Abacus mental arithmetic is taught multimodally, as both visual and motor operations. The abacus uses a finite set of rules or algorithms for moving the abacus beads to perform addition or subtraction of single digits (Supplemental Materials 3: How to Use an Abacus and Example Problems with Gesture Solutions). As Stigler et al. (1986) has pointed out, any arithmetic problem can be solved on an abacus using a fixed sequence of these algorithms. In many abacus schools, these algorithms for moving the beads are taught as stylized two-handed movements using the thumbs and index fingers held over two columns of beads for each operation (Supplemental Materials 1: Abacus Gestures 1–70). Teachers often correct students on the proper form of these hand movements using an abacus as instructional pedagogy (Supplemental Materials 2: The Abacus Hand Movement Lexicon and Correct and Incorrect Gestures). This manner of instruction is similar to how a pianist might learn proper hand positioning and fingering.

Although mental arithmetic is practiced at all stages, learners are allowed to use different physical aids during training. In a beginning abacus class, children primarily learn by manipulating a physical abacus using correct hand movements. An abacus is an array of beads with five beads in each column. There is a single upper row of beads separated by a horizontal bar from four lower rows of beads. When a bead in the upper row is pushed downward with the index finger to touch the horizontal bar, it registers a digit value of five for that column. When one of the beads in the four lower rows is pushed upward with the thumb to touch the horizontal bar, it registers a digit value of one for that column. Hence, each column can register a digit value from 0 to 9. When a column is designated as the one’s column (×10^0^), each successive column to the left is a successive power of ×10^n^ and each column to the right a successive negative power of ×10^-n^. Any arithmetic problem can be solved by concatenating a fixed sequence of these hand movements to move the beads according to algorithms for complements of 5 and 10 (see **Supplementary Materials 3** for more details).

As a transitional or intermediate stage of instruction, children use a picture card or static diagram of an abacus. Instead of moving beads on a physical abacus, learners touch the picture card or spontaneously gesture over it. This reduces the physical tool to an abstract mathematical diagram or sign. At the advanced stage, learners solve problems purely mentally without a physical abacus or any visual aid. However, as problem size and complexity of operations increase, learners may revert back to using one of the physical aids.

Learners at all stages of instruction, especially at intermediate and advanced performance levels, spontaneously produce co-thought gestures which closely mimic the hand movements when using a physical abacus. These movements can sometimes be exaggerated or vary in form. It is important to note that teachers instruct learners on the proper hand movements when using a physical abacus; but they do not instruct learners on how to spontaneously gesture. The only criterion is to solve the problems mentally. Thus, it is totally the learners’ decision to gesture or not. Overall, as abacus learners acquire mental arithmetic skill, they rely less on a physical abacus to perform mental arithmetic. They gradually internalize or embody the abacus tool. Whether and how these spontaneous co-thought gestures facilitate mental arithmetic remains unclear.

### The Present Study

The present study examined whether the beneficial effect and form of abacus co-thought gestures are different among beginning, intermediate, and advanced learners who are asked to solve one-, two-, and three-digit arithmetic problems. We tested the abacus mental arithmetic performance of 6- to 8-year-old beginning, intermediate, and advanced learners. Each learner was randomly assigned to one of three conditions for performing calculations at three difficulty levels (one-digit, two-digit, and three-digit): physical abacus, hands-free (spontaneous gesture) mental arithmetic, and hands-restricted mental arithmetic.

Based on the current state of research, there are several competing predictions about how abacus co-thought gestures may benefit learners at different skill levels when solving problems of varying degrees of difficulty. According to Image Maintenance theory, co-thought gestures, as bodily acts, should refresh the mental image of an abacus on a “visuospatial scratchpad” (Wesp et al., 2001, p. 592) and have a beneficial effect for learners at all levels of skill and problem difficulty. Moreover, according to Chu and Kita’s action generation hypothesis, abacus co-thought gestures should become increasingly representational and abstract as learners internalize the action strategy as they become more advanced in skill. Spontaneous gestures, in other words, would be less mimetic of physically manipulating an abacus. This would mean more advanced learners should have less accurate movements compared to beginning learners. However, neither the Image Maintenance Theory nor the action generation hypothesis are clear about the underlying mechanism by which gestures should have a beneficial effect.

In contrast, we hypothesize that abacus co-thought gestures facilitate mental arithmetic as motor programs that complement visual-spatial representation to reduce working memory load. We predict that abacus co-thought gestures will be beneficial only for learners who have acquired motor skills that closely reflect simulated action on a physical abacus. In other words, contrary to Image Maintenance theory, we predict that the beneficial effect of spontaneous abacus gestures will vary depending on skill level. And, contrary to the action generation hypothesis, more advanced learners’ spontaneous gestures will more accurately mimic action on a physical abacus.

According to GSA theory, spontaneous gestures activate motor programs that assist working memory as imagery tasks pass beyond a threshold of difficulty. As Beilock et al. (2004b) has noted, motor skills operate largely outside of working memory and thus reduce working memory load. This is based on Fitts and Posner (1967) proposal that working memory load is greatest at beginning stages of learning a motor skill because movements must be closely monitored and are often prone to error. With practice, these movements become increasingly automated as motor programs that require little conscious effort to produce increasingly accurate movements.

Additionally, numerous studies have shown that motor-spatial representation complements visual-spatial representation by encoding visual-spatial sequences as motor plans to reduce working memory load (Sirigu and Duhamel, 2001; Wymbs et al., 2012; Langner et al., 2014; Smithson and Nicoladis, 2014). Such motor-spatial representation, takes longer to acquire than visual-spatial representation. Bapi et al. (2000) and Hikosaka et al. (2002) have shown that in visual-motor tasks, learners acquire the sequence of visual-spatial coordinates faster than motor-spatial coordinates. However, after a motor sequence is learned, learners can execute them faster and with little conscious effort. A recent EEG and fMRI case study by Ku et al. (2012) has shown that abacus mental arithmetic may involve two parallel cortical loops, first activating one for visual-spatial processing, shortly followed by a second for motor-spatial processing (see also Hikosaka et al., 1999, 2002).

We thus expected that abacus learners would master how to use a physical abacus before becoming proficient in using abacus co-thought gestures. This is because being able to see the beads on a physical abacus as a visual-spatial sequence may demand less working memory compared to maintaining a motor-spatial mental representation of it. Beginning learners should be able to perform calculations well with a physical abacus. However, without the aid of a physical abacus, they would perform poorly under the spontaneous gesture condition and hands-restricted condition. Moreover, the accuracy of their gesturing would be poor because they had not yet acquired the motor-programs for abacus gestures.

Intermediate learners would perform mental calculations equally well using spontaneous gestures compared to using a physical abacus for simple one- and two-digit problems. Intermediate learners’ gestures should also be highly accurate, following closely the same types of hand movements for moving beads on a physical abacus. In other words, intermediate learners would have acquired motor-programs for abacus gestures to aid in visual-spatial representation of the mental abacus. However, as the demands on working memory increase with problem difficulty, the beneficial effect of co-thought gestures in reducing working memory load should attenuate for the most difficult problems. Intermediate learners, may thus perform less well using spontaneous gestures compared to physical abacus for more difficult three-digit problems. Moreover, the beneficial effect of co-thought gestures should be most salient when comparing intermediate learners’ ability to perform mental arithmetic under the hands-free spontaneous gesture to that in the hands-restricted condition. These learners would perform poorly in the hands-restricted condition because they had not yet fully automated and internalized the motor programs for abacus gestures to maintain the mental representation of an imaginary abacus.

Different from beginning and intermediate learners, advanced learners would have fully internalized and automated abacus gesture motor-programs. Thus, abacus co-thought gestures should not only have a beneficial effect for mental arithmetic performance but also show increasing movement accuracy similar to manipulating a physical abacus. Hence, advanced learners should not even need overt gesturing to refresh motor-spatial mental representation. They would be able to perform mental arithmetic calculations without much conscious effort through the assistance of gestures. Advanced learners’ gestures should be highly accurate and their calculation scores in the hands-free and hands-restricted conditions should be comparably high and nearly as high as with a physical abacus.

Evidence for these predictions comes from previous abacus studies. Hatano et al. (1977) tested 10 skilled abacus users and found that prohibiting hand movements and finger tapping similarly reduced performance for most subjects, but did not prevent the two most advanced subjects from correctly answering nearly all the problems. Likewise, Frank and Barner (2011) found that finger drumming significantly interfered with performance (p. 7); however, they noted that the most advanced participants were not affected. In a recent study, Brooks et al. (2017), found that advanced learners performed far worse with motor interference. These findings suggest that the role of gestures may vary for abacus learners at different skill levels, similar to our predictions for intermediate and advanced learners.

Understanding how the beneficial effect of abacus gestures changes at different levels of skill and problem difficulty can provide us with insights into the role of visual and motor working memory in abacus mental arithmetic. Some studies have shown that advanced abacus learners perform significantly better on mental arithmetic tasks compared to untrained controls (Lee et al., 2007; Chen et al., 2011). Such studies claim that mental abacus training focuses on visual working memory, resulting in improved calculating performance. In contrast, Frank and Barner (2011) have shown that mental arithmetic skill is not necessarily attributable to enhanced perceptual ability. They found that abacus experts and untrained controls performed similarly on a visual working memory task in which participants estimated the number of dots on flashcards. The study found that abacus experts perform fast mental calculations by employing a strategy of grouping columns of abacus beads to optimize visual working memory. Barner et al. (2016) likewise found participants’ differences in spatial working memory affected their individual ability to perform mental arithmetic, but did not change basic cognitive abilities, such as increasing number span.

Neurophysiological studies of abacus mental arithmetic show activation in cortical areas important for both visual and motor imagery. Activation occurs in the parietal cortex (Hanakawa et al., 2003) important for integrating visuospatial and motor input from the hands, as well as in the premotor cortex and Supplementary Motor Area (Chen C.L. et al., 2006; Chen F.Y. et al., 2006). Premotor cortex is important for motor planning and preparation of correct or incorrect movement. Supplementary Motor Area is important for movement sequence from memory and mental rehearsal of movement sequences. These studies have also shown that abacus experts compared to non-experts have reduced demands on frontal-subcortical areas related to the global workspace of executive function (Chen C.L. et al., 2006; Chen F.Y. et al., 2006; Li et al., 2013). This is consistent with claims that both visual grouping strategies and motor learning may reduce demands on working memory.

The current study also sheds light on how gesture may assist in transitioning from concrete objects to mental representation when learning arithmetic. The use of concrete manipulatives has been a staple of early mathematics education for decades (Post, 1981). Bruner (1966) has proposed that mathematics instruction should proceed in three stages: enactive, iconic, and symbolic. In the enactive stage, multiple physical objects or manipulates assist learners to grasp mathematical concepts by providing a store of concrete or embodied real-world experiences. By comparing these multiple examples, learners in the iconic stage then strip away extraneous perceptual details to use graphical or pictorial representations. Lastly, learners in the symbolic stage extract abstract concepts, represented in formal notation.

Theoretical models such as Bruner’s for mathematics learning has been widely applied in curricula, especially for early childhood education (Fyfe et al., 2014). Among many notable examples are the Montessori use of concrete to abstract sensorial material such as beads, rods, and blocks for counting, arithmetic, and decimals; the Rational Number Project for teaching fractions; and the Concrete-Representational-Abstract sequence used by MathVIDS and other curricula for students struggling with basic concepts. Since the 1980s, the Concrete-Pictorial-Abstract method has remained a cornerstone of the Singapore Ministry of Education mathematics curricula (Leong et al., 2015).

Despite widespread implementation of the enactive-iconic-symbolic model in school curricula, little work has explored the mechanisms underlying how learners shift from embodied concrete perception and action to abstract concepts. This lack of explanation has further led to controversy over whether concrete manipulatives are even effective. Some studies have shown that in certain circumstances instruction with concrete manipulates led to worse performance (Kaminski et al., 2008). Others have noted that there may be many mechanisms underlying how learners transition from concrete objects to symbolic representation (McNeil and Fyfe, 2012).

## Materials and Methods

### Participants

One hundred and eighty children (half males) participated in this experiment from 2010 to 2011. They were English speaking Singaporeans and attended abacus classes at the Classical Mental Arithmetic School (CMA). CMA was one of the popular schools in Singapore teaching young learners abacus mental arithmetic using two hands and four fingers. It has 21 branches in Singapore. In the present study, we collected data in five of them. On average, children were 7;1 (years;months) years old, ranging from 5;11 to 8;1 years old. All of them were typical primary school students. Abacus training is common among Singaporean children, across socioeconomic and educational backgrounds. The selected participants were thus a representative sample. We further note that children at this young an age are at the very beginning stage of their mathematics education in primary school. We chose a narrow age range to minimize the influence of the students’ regular school education. All the procedures were approved by the institutional review board of the authors’ university at the time of the study, in compliance with the Declaration of Helsinki. We obtained the parents’ informed consent prior to the study. The first author presented preliminary work for this article at the workshop, Culture and Cognition in Asia II: Performative Gesture in Religion and Science (17 June 2010 at the National University of Singapore, https://ari.nus.edu.sg/Event/Detail/1066).

### Design and Procedures

Each child was classified into one of the following three categories: beginning learners (*N* = 57), intermediate learners (*N* = 65), and advanced learners (*N* = 54), based on the CMA program in which the child was enrolled. CMA classifies students into beginning, intermediate, and advanced levels according to a series of finely-grained examinations. Students progress through a series of exercise workbooks in which numbers are aurally presented on CD and must be added or subtracted mentally. These exams neither require students to gesture nor test their movement accuracy. Learners progress through exercises, beginning with 2 one-digit numbers up through 10 one-digit numbers; this is followed by 2 two-digit numbers up through 10 two-digit numbers. At the beginning of each class, students are tested and must score perfectly before being allowed to proceed. Beginning learners must be able to mentally calculate from four one-digit numbers up to three two-digit numbers. Intermediate learners must be able to mentally calculate from four two-digit numbers up to three three-digit numbers. Advanced learners must be able to mentally calculate from four three-digit numbers and above.

We then randomly assigned learners from each skill level to one of the following three conditions: (1) physical abacus; (2) hands-free mental arithmetic (spontaneous gesture); (3) hands-restricted mental arithmetic. **Table 1** shows the demographic information of participants in all conditions.

**Table 1:** Demographic information of participants in all conditions.

	Physical Abacus			Hands-Free Mental Arithmetic			Hands-Restricted Mental Arithmetic		
	Beginning (*n* = 19)	Intermediate (*n* = 20)	Advanced (*n* = 20)	Beginning (*n* = 19)	Intermediate (*n* = 28)	Advanced (*n* = 19)	Beginning (*n* = 15)	Intermediate (*n* = 22)	Advanced (*n* = 21)
Mean	6;11	6;11	7;2	6;11	7;2	7;1	7;1	7;2	6;10
Age (year; month)	*SD* = 0.73	*SD* = 0.57	*SD* = 0.58	*SD* = 0.60	*SD* = 0.53	*SD* = 0.56	*SD* = 0.62	*SD* = 0.60	*SD* = 0.68
Gender (male)	8	12	11	9	15	10	8	13	10


We refer to mental arithmetic as MA. Learners in all conditions were tested individually at their CMA branch and asked to solve 60 addition and subtraction questions (20 one-digit, 20 two-digit, and 20 three-digit). All questions were designed by teachers in CMA. Learners were given 30 min to complete the test, which was ample time for all to finish the problems that they were able to do. However, learners who found that the problems were too difficult to manage could stop at any time. The entire experiment was videotaped. Each child was closely monitored by an experimenter, one-on-one, for compliance. None of the children moved his/her hands in the hands-restricted mental arithmetic condition.

Learners in the physical abacus condition solved the problems using a physical abacus, which was the same as the one they used in their regular class. Learners in the hands-free MA (spontaneous gesture) condition solved the same problems, but without the assistance of an abacus. With prompting, they were able to spontaneously move their hands to perform mental calculations. Learners in the hands-restricted MA condition also solved the same problems using mental calculation, but were restricted from moving their hands by holding a ball with both hands.

### Scoring and Coding

We calculated the mean proportions of questions with correct answers, which were calculated as the number of correct answers separately divided by the total number of questions at each digit-level in each group of learners in each condition.

A teacher at CMA then coded the abacus hand movements and abacus gestures produced by learners in the physical abacus condition and hands-free mental arithmetic condition, respectively. Teachers at CMA were well trained in identifying the abacus hand movements and gestures produced by their students. There are two kinds of hand movements: abacus hand movements, produced when manipulating a physical abacus; and abacus hand gestures, produced while doing mental calculation. The teacher coded both kinds of hand movements using a standard answer key, which provided the sequence of gestures to solve each problem. Supplemental Materials 1: Abacus Gestures 1–70, provides a list of illustrations for each of the 70 two-handed gestures and the arithmetic operations they perform. Supplemental Materials 2: The Abacus Hand Movement Lexicon and Correct and Incorrect Gestures, provides the basic 16 hand movements (including no movement) using the left and right index fingers and thumbs. These movements are combined to form the 70 abacus gestures. Supplemental Materials 2 also has examples of correct and incorrect gestures. Supplemental Materials 3: How to Use an Abacus and Example Problems with Gesture Solutions, explains of how to use an abacus and gives examples of one-digit and two-digit problems with the sequence of gestures to compute the answers. We counted the number of abacus hand movements or abacus hand gestures produced per question.

After identifying a gesture, the teacher determined whether the gesture was correct. We sought to understand how learners at different skill levels employed correct gestures or other movements in mental calculations and how the sequence of these correct gestures compared to hand movements when manipulating a physical abacus. Learners are taught in abacus classes stylized or pedagogically correct hand movements using the index finger and thumb up or down in a single column, either as one hand or as two hands in adjacent columns. When learners perform mental arithmetic, they often spontaneously gesture in the air, mimicking these hand movements to move the beads on physical abacus. Each gesture has specific algorithmic meaning depending on context and is executed in a fixed sequence of gestures to solve a particular mathematical problem. However, learners sometimes do not use these correct hand movements or gestures and make mistakes, such as incorrectly moving their index fingers and thumbs, skipping or combining movements, or using fingers other than the index fingers and thumbs. We compared the proportion of correct gestures produced in the hands-free MA (spontaneous gesture) condition to the proportion of correct hand movements in the physical abacus condition. The proportion of correct hand movements or gestures was calculated as the total number of correct hand movements or gestures divided by the total number of fixed algorithmic steps.

### Inter-coder Reliability

To assess inter-coder reliability for the coding of the abacus hand movements/gestures and that of the correct gestures, we randomly selected twelve children (three in each condition) for independent coding by a second trained coder. The coder was also one of the teachers at CMA and she was naive to our hypotheses. The inter-rater agreement was 0.96 (*N* = 5580; Cohen’s kappa = 0.94, *p* < 0.001) for the coding of the number of abacus hand movements. The inter-rater agreement was 0.92 (*N* = 4680; Cohen’s kappa = 0.94, *p* < 0.001) for the coding of the abacus hand gestures. As for the coding of the accuracy of the correct abacus hand movements, the inter-rater agreement was 0.88 (*N* = 5357; Cohen’s kappa = 0.84, *p* < 0.001). With regard to the coding of the accuracy of the correct abacus hand gestures, the inter-rater agreement was 0.85 (*N* = 4305; Cohen’s kappa = 0.82, *p* < 0.001).

## Results

We examined whether the facilitating role of gesture in solving arithmetic problems varied with the level of abacus skills and the difficulty of problems. We first examined how learners with different levels of abacus skills gestured, by looking at whether these gestures were correct, i.e., following the form of hand movements on an abacus taught in class. We next examined the proportions of correct answers. We investigated these proportions as functions of the method of calculation, level of abacus skills of learners, and level of problem difficulty. The accuracy rate, as the proportion of questions answered correctly, was calculated as the total number of questions answered correctly divided by the total number of questions.

Abacus hand movements produced in the physical abacus condition and abacus gestures produced in the mental arithmetic condition were classified into two categories: correct and incorrect. The proportion of correct abacus hand movements or abacus gestures was calculated as the total number of correct abacus hand movements divided by the total number of abacus gestures possibly produced.

**FIGURE 1:**
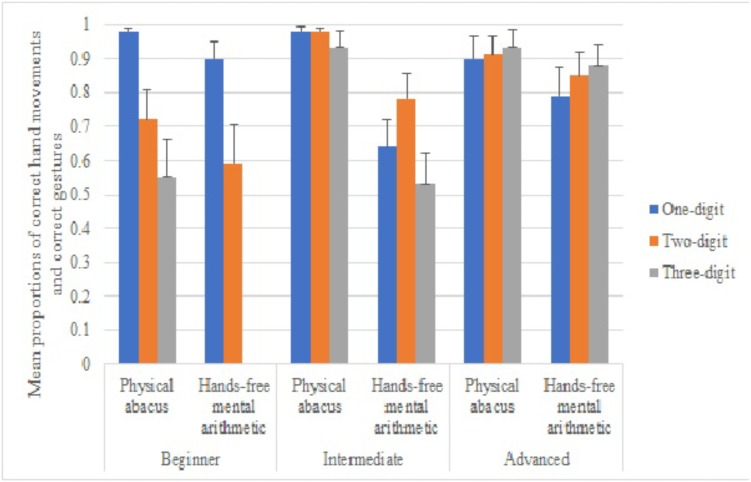
Mean proportions of correct hand movements in the physical abacus condition and correct gestures in the hands-free mental arithmetic condition for one-, two-, and three-digit problems at beginning, intermediate and advanced skill-levels.

**Figure 1** shows the proportions of correct abacus hand movements or abacus gestures produced in the physical abacus and the mental arithmetic conditions. We ran a repeated measures ANOVA with the difficulty of problems as the independent within-subject variable, condition and skill level as the independent between-subject variables, and the proportion of correct abacus hand movements or abacus gestures as the dependent variable. We found a significant effect for the problem difficulty, *F*(2,194) = 135.13, *p* < 0.001, partial η^2^ = 0.58, condition, *F*(1,97) = 32.29, *p* < 0.001, partial η^2^ = 0.25, skill level, *F*(2,97) = 7.97, *p* < 0.001, partial η^2^ = 0.14, skill level and condition interaction, *F*(2,97) = 3.25, *p* = 0.043, partial η^2^ = 0.06, problem difficulty and skill level, *F*(4,194) = 12.06, *p* < 0.001, partial η^2^ = 0.20, problem difficulty and condition, *F*(2,194) = 29.05, *p* < 0.001, partial η^2^ = 0.23, and three–way interaction, *F*(4,194) = 3.15, *p* = 0.02, partial η^2^ = 0.06. The *post-hoc* statistical power for this test with respect to alpha level of 0.05 was 0.95 (G^∗^Power 3.1.9.2; Erdfelder et al., 2005).

Given the significant three–way interaction, we separately looked at the differences in the proportions of correct abacus hand movements or abacus gestures produced in the physical abacus and the mental arithmetic conditions among three groups of learners. As for beginning learners, we found a significant effect for the problem difficulty, *F*(2,70) = 46.05, *p* < 0.001, partial η^2^ = 0.57, condition, *F*(1,35) = 11.19, *p* < 0.001, partial η^2^ = 0.89, and interaction, *F*(2,70) = 6.83, *p* = 0.002, partial η^2^ = 0.16. Bonferroni adjusted-pairwise comparisons showed that beginning learners produced correct abacus hand movements more often in one-digit questions than in two-digit questions, *p* < 0.001, and three-digit questions, *p* < 0.001, in the physical abacus condition. However, there was no significant difference between two- and three-digit questions, *p* = 0.53. In the mental arithmetic condition, they produced correct abacus gestures in one- and two-digit questions more often than in three-digit questions, *ps* < 0.001. As for intermediate learners, we found a significant effect for the problem difficulty, *F*(2,92) = 3.80, *p* < 0.030, partial η^2^ = 0.08, and condition, *F*(1,46) = 18.54, *p* < 0.001, partial η^2^ = 0.29. The interaction was not significant, *F*(2,92) = 1.75, *p* < 0.180. Interestingly, they marginally produced more correct abacus hand movements or gestures when solving two-digit than one-digit and three-digit problems, *ps* < 0.060. They produced more correct abacus hand movements or gestures in the physical abacus condition than in the mental arithmetic condition, *p* = 0.001. As for advanced learners, we found no significant effects for the problem difficulty, *F*(2,76) = 0.30, *p* = 0.74, partial η^2^ = 0.03, condition, *F*(1,38) = 1.09, *p* = 0.30, partial η^2^ = 0.03, and interaction, *F*(2,76) = 0.31, *p* = 0.740, partial η^2^ = 0.008. It suggested that advanced learners were capable in producing correct gestures or hand movements in all three different levels of problem difficulty and in two different conditions.

**FIGURE 2:**
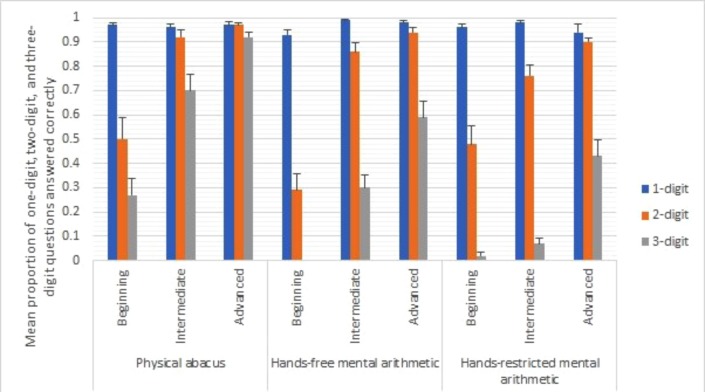
Mean proportions of questions answered correctly in one-, two- and three-digit questions for beginning, intermediate and advanced learners in three conditions: physical abacus, hands-free mental arithmetic, and hands-restricted mental arithmetic.

We next examined the proportions of one-digit, two-digit, and three-digit questions answered correctly in three groups of learners in three different conditions. **Figure 2** shows the performance of learners.

We ran a repeated measures ANOVA with the difficulty of problems as the independent within-subject variable, condition and skill level as the independent between-subject variables, and the proportion of questions answered correctly as the dependent variable. We found a significant effect for the problem difficulty, *F*(2,318) = 437.44, *p* < 0.001, partial η^2^ = 0.73, condition, *F*(2,159) = 44.28, *p* < 0.001, partial η^2^ = 0.36, skill level, *F*(2,159) = 46.78, *p* < 0.001, partial η^2^ = 0.37, skill level and condition interaction, *F*(4,159) = 3.21, *p* = 0.014, partial η^2^ = 0.08, problem difficulty and skill level, *F*(4,318) = 26.65, *p* < 0.001, partial η^2^ = 0.25, problem difficulty and condition, *F*(4,318) = 41.57, *p* < 0.001, partial η^2^ = 0.34, and three–way interaction, *F*(8,318) = 2.71, *p* = 0.007, partial η^2^ = 0.06. The *post-hoc* statistical power for this test with respect to alpha level of 0.05 was 0.95 (G^∗^Power 3.1.9.2; Erdfelder et al., 2005). Because of the presence of the three–way interaction, we ran separate repeated measures ANOVA for different groups of learners. For beginning learners, there were significant effects for problem difficulty, *F*(2,100) = 294.50, *p* < 0.001, partial η^2^ = 0.86, condition, *F*(2,50) = 5.71, *p* = 0.006, η^2^ = 0.19, and interaction, *F*(4,100) = 3.49, *p* = 0.010, partial η^2^ = 0.12. The proportion of one-digit and two-digit questions answered correctly was comparable across different conditions, one-digit: *F*(2,50) = 2.90, *p* = 0.06; two-digit: *F*(2,50) = 2.40, *p* = 0.10, although participants scored the lowest in the mental arithmetic condition for the two-digit problems. However, there was significant difference in the three-digit questions, *F*(2,50) = 12.36, *p* < 0.001. Bonferroni adjusted-pairwise comparisons showed that the proportion of three-digit questions answered correctly was greater in the physical abacus condition than in the mental arithmetic condition, *p* < 0.001, and in the hand movements prevented condition, *p* < 0.001. However, there was no difference between mental arithmetic and hand movements prevented conditions.

For intermediate learners, there were significant effects for problem difficulty, *F*(2,134) = 310.68, *p* < 0.001, partial η^2^ = 0.82, condition, *F*(2,67) = 21.15, *p* < 0.001, partial η^2^ = 0.39, and interaction, *F*(4,134) = 26.47, *p* < 0.001, partial η^2^ = 0.44. The proportion of one-digit questions answered correctly was comparable across different conditions, *F*(2,67) = 2.38, *p* = 0.10. However, there were significant differences in the two- and three-digit questions, two-digit: *F*(2,67) = 4.11, *p* = 0.021; three-digit: *F*(2,67) = 35.08, *p* < 0.001. Bonferroni adjusted-pairwise comparisons showed that the proportion of two-digit questions answered correctly was greater in the physical abacus condition than in the hand movements prevented condition, *p* = 0.020. There was no significant difference between the physical abacus condition and the mental arithmetic condition, and between the mental arithmetic condition and the hand movements prevented condition, although participants in the mental arithmetic condition tended to perform better than those in the hand movements prevented condition. The proportion for the three-digit questions answered correctly was greater in the physical abacus condition than in the mental arithmetic condition, *p* < 0.001, and in the hand movements prevented condition, *p* < 0.001. The proportion was also higher in the mental arithmetic condition than in the hand movements prevented condition, *p* < 0.005.

The findings in the advanced learners were similar to those in the intermediate learners. There were significant effects for problem difficulty, *F*(2,114) = 91.86, *p* < 0.001, partial η^2^ = 0.62, condition, *F*(2,57) = 18.25, *p* < 0.001, partial η^2^ = 0.39, and interaction, *F*(4,114) = 17.13, *p* < 0.001, partial η^2^ = 0.38. The proportion of one-digit questions answered correctly was comparable across different conditions, *F*(2,57) = 0.99, *p* = 0.38. However, there were significant differences in the two- and three-digit questions, two-digit: *F*(2,57) = 4.23, *p* = 0.019; three-digit: *F*(2,67) = 21.77, *p* < 0.001. Bonferroni adjusted-pairwise comparisons showed that the proportion of two-digit questions answered correctly was greater in the physical abacus condition than in the hand movements prevented condition, *p* = 0.020. There was no significant difference between the physical abacus condition and the mental arithmetic condition, and between the mental arithmetic condition and the hand movements prevented condition, *p* = 0.25. The proportion for the three-digit questions answered correctly was greater in the physical abacus condition than in the mental arithmetic condition, *p* < 0.001, and in the hand movements prevented condition, *p* < 0.001. The proportion in the mental arithmetic condition was not different from that in the hand movements prevented condition, *ns*, although participants in the mental arithmetic condition tended to perform better than those in the hand movement prevented condition.

## Discussion

### Interpretations

Our results showed that the beneficial effect of abacus gestures on the accuracy of calculations varied with learners’ skill level and problem difficulty. There was a clear contrast in the gesturing behavior and calculation performance of learners at different skill levels. Learners first mastered how to calculate using a physical abacus and later benefitted from using abacus gestures, answering more questions correctly when allowed to gesture compared to not gesturing. This suggested that learners acquired the ability to calculate using visual-motor spatial sequence, as the arrangement of abacus beads, followed by motor-spatial sequence, as abacus gestures.

At each skill level, the differences between using a physical abacus, gestures, or no gestures also varied according to problem difficulty. The results indicated that as demands on working memory increased with problem difficulty, gestures assisted up to a point for mental arithmetic before learners resorted back to performing better on a physical abacus. Hand movement accuracy for especially intermediate and advanced learners also reflected motor learning. The difference in movement accuracy between the physical abacus and hands-free spontaneous gesture conditions showed a trend in increased movement accuracy following skill level; beginning learners had low movement and gesture accuracy while intermediate and advanced learners had high accuracy.

More specifically, beginning learners were able to perform calculations with a physical abacus even up to three-digit problems. However, they performed poorly in both hands-free and hands-restricted conditions to the point that at two-digit and three-digit problems, there was no significant difference between the two mental arithmetic conditions. This showed that beginners were able to correctly solve some difficult problems when able to see the arrangement of beads on a physical abacus, but did not benefit much from using gestures to manipulate an imaginary abacus for mental calculations. This pattern was also reflected in the poor accuracy of beginners’ hand movements. Movement accuracy was greatest with a physical abacus, especially for one-digit problems. But, under the hands-free mental arithmetic condition, gesture accuracy was equally poor for one-digit and two-digit problems, and nearly all inaccurate for three-digit problems. This clearly indicated that beginners had not yet learned how to calculate using gestures and still needed the aid of a physical abacus.

In contrast, gestures facilitated problem solving for intermediate learners in the hands-free condition, compared to both the physical abacus and hands-restricted conditions. The trend showed that one-digit problems were simple enough for intermediates to perform equally well in all conditions. At two-digit problems, intermediates could calculate just as well using gestures as with a physical abacus, but not when their hands were restricted. By three-digit problems the contrast was even clearer. Intermediates performed best with a physical abacus, indicating that intermediate learners’ ability to use gestures assisted only up this point. Yet notably, at three-digit problems, intermediates performed mental arithmetic significantly better when allowed to gesture compared to when their hands were restricted from moving. This clearly showed that intermediate learners had gained the ability to successfully use gestures as well as a physical abacus up to two digits, but not three digits. And, when the demands on working memory were highest at three-digit problems, gestures had a beneficial effect compared to not gesturing during mental arithmetic.

This beneficial effect of gestures also seemed to be related to movement accuracy. Overall, intermediates’ movement accuracy with gestures was almost as high as with a physical abacus. This trend continued for advanced learners, whose hand movement accuracy was just as high with spontaneous gestures as with a physical abacus. Interestingly, intermediates’ movements were significantly more accurate at two-digit problems compared to simpler one-digit problems or more difficult three-digit problems.

Studies of motor-skill learning and automaticity have shown that novice and intermediate learners perform better under conditions for online-attentional monitoring of their movements, while advanced learners perform better when explicit attentional control is prevented (Beilock et al., 2004a). Intermediates may have had more accurate movements at two-digit problems compared to one-digit problems because they paid closer attention to their movements for the more difficult task. Moreover, their gesture accuracy declined at three digits compared to two digits because, as noted earlier, two digits was the threshold at which intermediates could use gestures as well as a physical abacus. The pattern of intermediate learners’ calculation score and movement accuracy thus reflected motor learning. Intermediate learners had acquired the basic motor programs of abacus gestures and were able to apply gestures more reliably and effectively, but had not yet reached full automaticity in their movements.

Advanced learners showed a mastery of mental arithmetic even without the use of gestures or a physical abacus. At two digits, advanced learners performed equally well using just gesture compared to using a physical abacus. In contrast to intermediate learners, advanced learners performed equally well at three digits in the hands-free and hands-restricted conditions. This indicated that advanced learners could use and maintain a mental representation of the abacus even without gesture. In contrast to beginning and intermediate learners, advanced learners gestural movements were highly accurate, regardless of problem difficulty. This indicated a higher degree of motor automaticity and internalization of the abacus representation for advanced learners compared to intermediate and beginning learners.

### Theoretical Implications

These contrasts among beginning, intermediate, and advanced learners in calculation performance and movement accuracy support the interpretation that abacus co-thought gestures are learned as a motor-skill that complements visual-spatial mental representation. A growing body of research shows that motor and visual imagery are complementary processes (Sirigu and Duhamel, 2001). Langner et al. (2014) has shown how the encoding of visual-spatial sequences of dots on the fingers of a schematic hand, translated working memory into sequential motor action. Recall was less accurate for longer sequences, but initiated faster after long delays. An fMRI analysis showed that activation in motor areas, especially basal ganglia, predicted recall after long delays. This indicated that visual spatial sequences were encoded as motor plans, possibly reinforced through mental rehearsal. Similar conversion of visual working memory to motor sequence has been shown in intracranial single-neuron studies of monkey premotor cortex (Ohbayashi et al., 2003). Smithson and Nicoladis (2014) have shown that iconic gesture production facilitates visual-spatial working memory activation during complex visual distractor inference.

Additional behavioral and fMRI studies have demonstrated that visual-spatial and motor-spatial sequences are acquired at different rates and skill levels. Bapi et al. (2000, 2006) found that beginning learners on a square grid key-pressing task quickly acquired visual-spatial sequence as coordinates on a rotated visual display. Spatial sequence is first acquired as it is effector-unspecific, but requires maximum attention or working memory. However, intermediate and advanced learners concurrently learned the visual and motor sequence as motor coordinates on a rotated input keypad. Moreover, they showed significant reduction in reaction-time under the motor compared to visual conditions. This indicated that the motor sequence was slower to acquire but quicker to perform once mastered.

Hikosaka et al. (1999, 2002) has proposed two parallel cortical systems that independently code visual and motor coordinates. Visuospatial representation forms a loop between frontoparietal cortex with the associative regions of basal ganglia (anterior striatum) and cerebellum. Motor representation links the Supplementary Motor Area with the motor regions of basal ganglia (posterior striatum) and cerebellum. Wymbs et al. (2012) suggests that Hikosaka’s motor loop may be related to the concatenation of chunks (p. 934) and found increased fMRI BOLD activity in the bilateral putamen of the basal ganglia during the concatenation of motor schemas. Modular Selection and Identification Controller (MOSAIC) theory stipulates that multiple internal models of novel tools are acquired and modularly organized in the cerebellum. After learning an internal model in the cerebellum, the output is sent to premotor regions (Tamada et al., 1999). Imamizu et al. (2000) have further shown that after short but intensive training on a rotated joystick task, cerebral cortex activation decreased in the prefrontal and parietal regions but increased in the premotor and Supplementary Motor Area. We suggest that abacus training may provide an additional experimental paradigm for further research on multimodal representation in the cerebellum and basal ganglia.

Analogous to grouping strategies for visual working memory, motor sequences can be grouped or “chunked” as gestures. Wymbs et al. (2012) has shown that visual-spatial sequences can be concatenated as motor-spatial “chunks,” which are executed as a series of schemas. Inspired by musical notation, Wymbs developed a visual-motor sequence task using four fingers on one hand to show that “chunking” of individual movements into a single motor schema reduces the memory load during performance. Chunking forms hierarchical memory structures to support increased speed and accuracy during performance. Single motor schema can be concatenated into a series of motor schemas as longer operations.

It is possible that correct abacus gestures form chunked motor sequences representing arithmetic operations, thereby facilitating mental calculations. Once acquired as motor-chunks, learners are then able execute combinations of these gestures in a series to perform more complex calculations. Such conceptual and motor chunking may reduce cognitive load. Skilled learners, who have acquired abacus gestures, thus need only to decide on which gesture to execute, given the arrangement of beads. This reduces working memory load when calculating because changing the arrangement of beads is executed as a motor sequence. While it takes time to learn how to use gestures to do mental arithmetic without the visual assistance of a physical abacus, advanced learners can execute the calculation easily once they have acquired the learned motor-sequence.

Previous fMRI studies of abacus mental arithmetic have shown greater activation in non-experts compared to experts of frontal-subcortical areas related to the global workspace of executive function (Chen C.L. et al., 2006; Chen F.Y. et al., 2006). In contrast, experts had less activation of executive areas, but more involvement of right dorsal premotor cortex during mental calculation. This suggests that non-experts, who have not automated abacus gestures as motor-chunks, have a greater working memory load related to executive function. Experts, on the other hand, are able to execute each arithmetic operation without paying close attention to the operation’s physical execution. Additional studies of a variety of motor and higher cognitive tasks indicate that expert learners benefit most from mental rehearsal and imaginary practice (Cooper et al., 2001). Mental or covert rehearsal relies on limited working memory without the aid of an external tool like the abacus. Learners with a high level of prior knowledge or skill benefit most from mental practice because they have acquired schemas that free working memory. Experts are thus able to focus on rehearsing or automating these schemas and better able to combine them.

### Educational Applications and Future Directions

Recent studies of abacus mental arithmetic and task switching have found that abacus training also improves higher-order math abilities beyond basic arithmetic, multiplication, and division. Long-term learners perform significantly better than untrained peers on more abstract tasks including algebraic number filling (e.g., 4+_ = 3 + 7), number sequence recognition, numerical working memory, and visual-spatial counting and matching (Hu et al., 2011; Li et al., 2013; Wang et al., 2015). Hence, abacus gestures may promote learning not just as a physical action but support abstract representation (Novack et al., 2014). Spontaneous gestures may thus provide multimodal representations of number complements and relationships that allow learners to grasp complex calculations. This is consistent with previous non-abacus studies that have shown that spontaneous gestures generally aid in learning mathematics (Goldin-Meadow et al., 2009). Notably, our study focused on 6–8 year old children at an early stage of their mathematics education. It would be useful to test if spontaneous abacus gestures not only have a beneficial effect on mathematics performance but also the rate of mathematics learning.

## Conclusion

Abacus co-thought gestures have a clear beneficial effect for maintaining a mental representation of the abacus while performing mental arithmetic. These gestures are learned as specific movements using the index fingers and thumbs for moving abacus beads according to algorithms for complements of 5 and 10. Learners first acquire a basic skill in using a physical abacus and then acquire proficiency in using abacus gestures. The results indicate that this beneficial effect and accuracy of abacus gestures is related to motor learning. Beginners benefit little from using abacus gestures and their movement accuracy is poor. Intermediates perform mental arithmetic better when allowed to spontaneously gesture compared to when their hands are restricted. According the Gesture as Simulated Action theory, such spontaneous gestures are used when the demands of working memory reach a threshold. Advanced learners’ mental abacus score and gesture accuracy were comparatively high, regardless of whether they gestured or not. This indicates that they had automated the motor programs of abacus gestures. Such automated motor programs can be executed with little conscious effort or demand on working memory. These results are consistent with previous findings on mental arithmetic that found that learners at different skill levels improved in their use of visual strategies. Moreover, our findings suggest that abacus gestures act as motor programs that complement such visual-motor representation. This interpretation is supported by behavioral and neurophysiological studies which indicate that visual-spatial and motor-spatial learning are two complementary systems.

## Author Contributions

All authors listed have made a substantial, direct and intellectual contribution to the work, and approved it for publication.

## Conflict of Interest Statement

The authors declare that the research was conducted in the absence of any commercial or financial relationships that could be construed as a potential conflict of interest.
