# Supplementary material for: A Feel for Numbers: The Changing Role of Gesture in Manipulating the Mental Representation of an Abacus Among Children at Different Skill Levels
Source: Front Psychol. 2018 Aug 7;9:1267. doi: 10.3389/fpsyg.2018.01267 (PMC6090447; doi:10.3389/fpsyg.2018.01267)
Supplement: Supplementary file 1 [file Presentation_1.pdf]

Supplemental Materials 1

Abacus Gestures 1 - 70

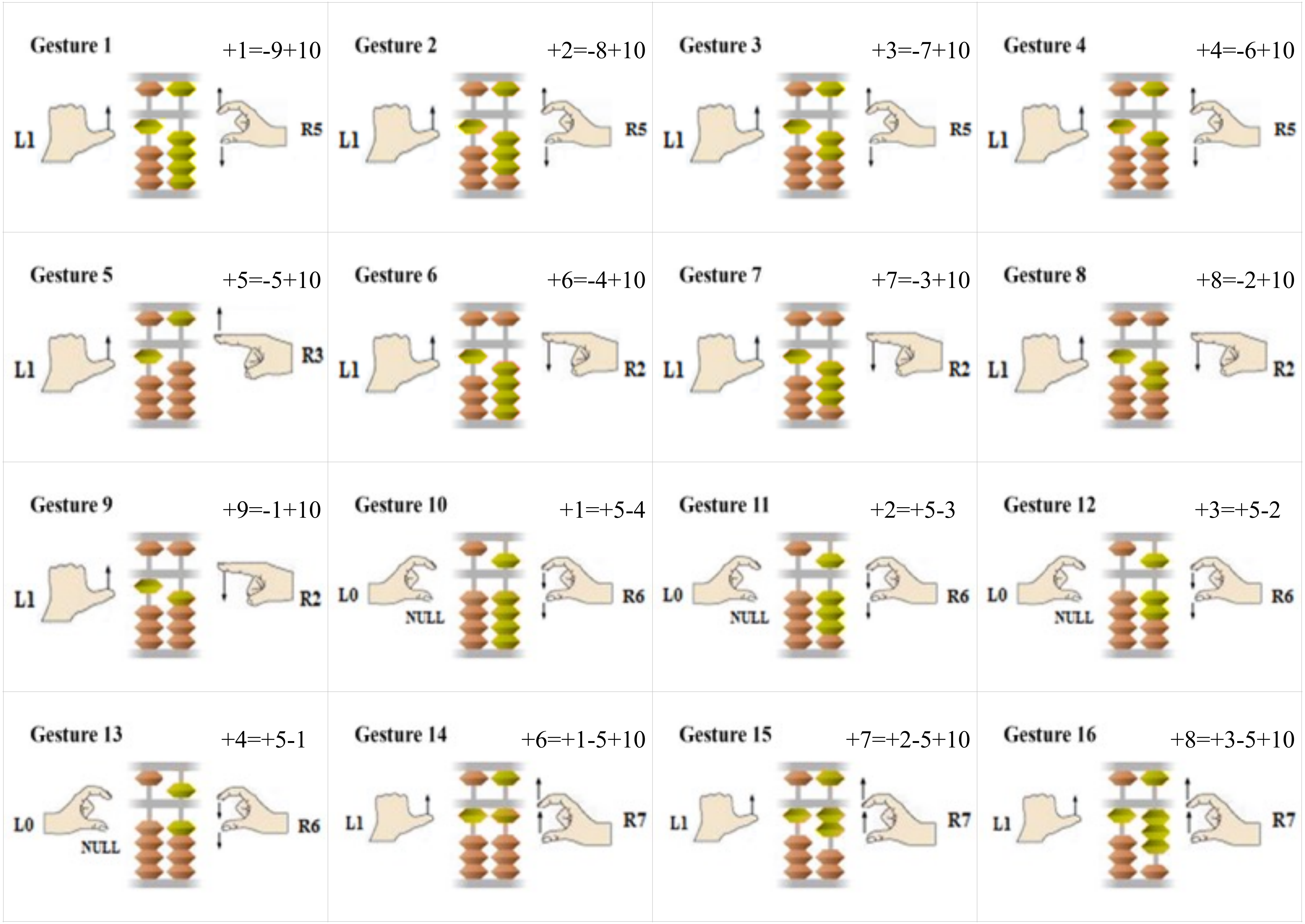

Gesture 1
+1=-9+10
L1
R5
Gesture 2
+2=-8+10
L1
R5
Gesture 3
+3=-7+10
L1
R5
Gesture 4
+4=-6+10
L1
R5
Gesture 5
+5=-5+10
L1
R3
Gesture 6
+6=-4+10
L1
R2
Gesture 7
+7=-3+10
L1
R2
Gesture 8
+8=-2+10
L1
R2
Gesture 9
+9=-1+10
L1
R2
Gesture 10
+1=+5-4
L0
NULL
R6
Gesture 11
+2=+5-3
L0
NULL
R6
Gesture 12
+3=+5-2
L0
NULL
R6
Gesture 13
+4=+5-1
L0
NULL
R6
Gesture 14
+6=+1-5+10
L1
R7
Gesture 15
+7=+2-5+10
L1
R7
Gesture 16
+8=+3-5+10
L1
R7

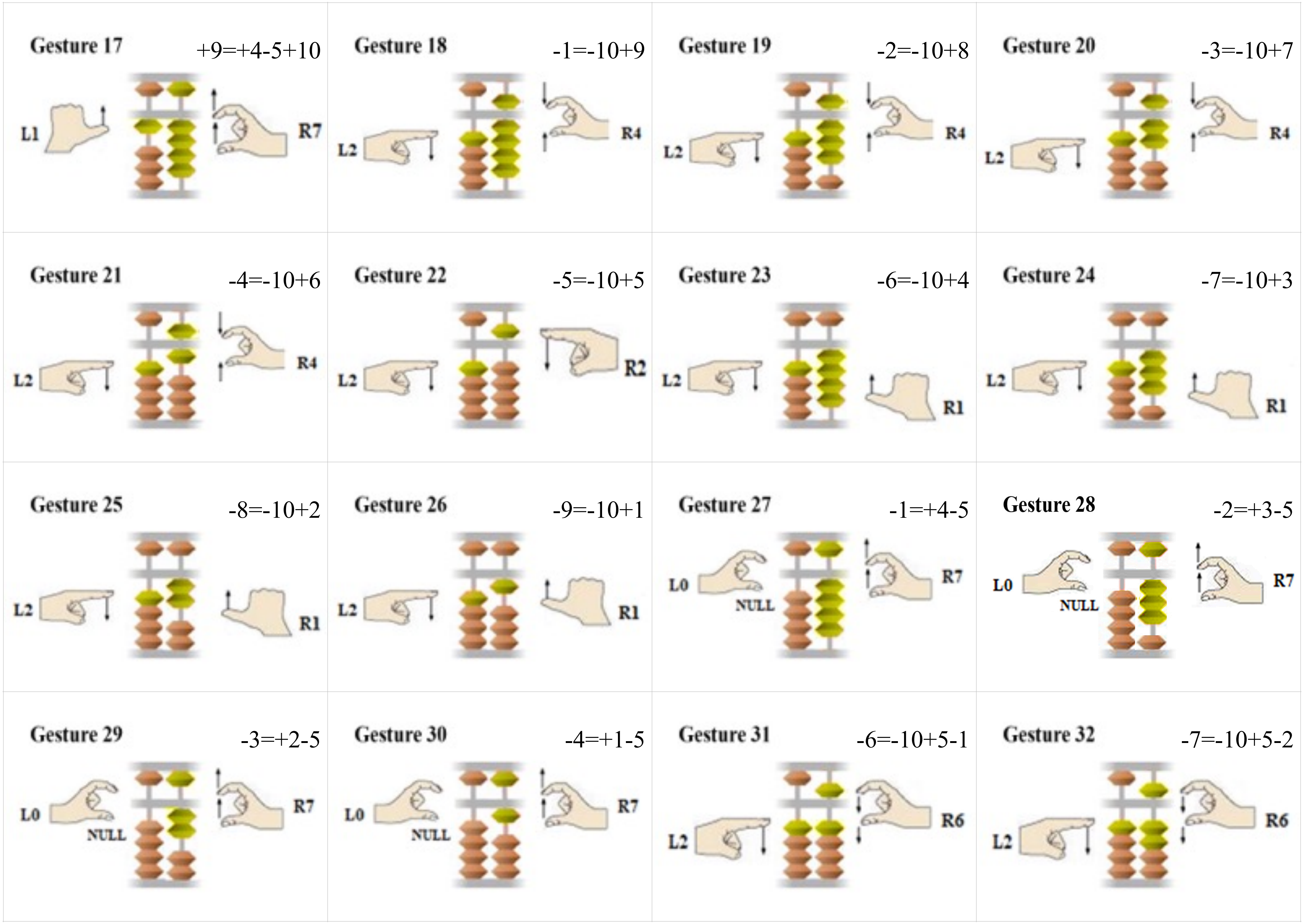

Gesture 17
+9=+4-5+10
L1
R7
Gesture 18
-1=-10+9
L2
R4
Gesture 19
-2=-10+8
L2
R4
Gesture 20
-3=-10+7
L2
R4
Gesture 21
-4=-10+6
L2
R4
Gesture 22
-5=-10+5
L2
R2
Gesture 23
-6=-10+4
L2
R1
Gesture 24
-7=-10+3
L2
R1
Gesture 25
-8=-10+2
L2
R1
Gesture 26
-9=-10+1
L2
R1
Gesture 27
-1=+4-5
L0
NULL
R7
Gesture 28
-2=+3-5
L0
NULL
R7
Gesture 29
-3=+2-5
L0
NULL
R7
Gesture 30
-4=+1-5
L0
NULL
R7
Gesture 31
-6=-10+5-1
L2
R6
Gesture 32
-7=-10+5-2
L2
R6

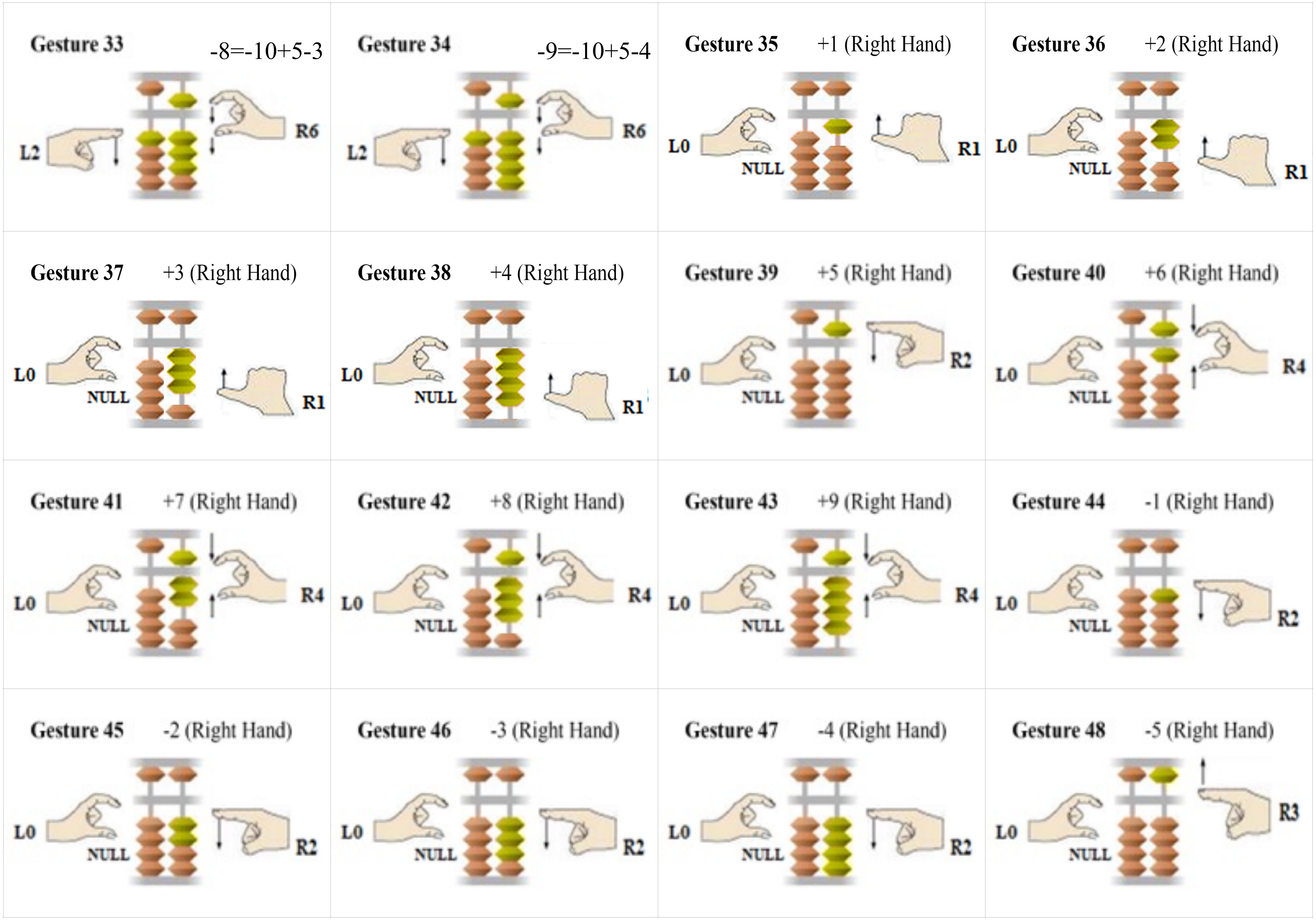

Gesture 33
-8=-10+5-3
L2
R6
Gesture 34
-9=-10+5-4
L2
R6
Gesture 35
+1 (Right Hand)
L0
NULL
R1
Gesture 36
+2 (Right Hand)
L0
NULL
R1
Gesture 37
+3 (Right Hand)
L0
NULL
R1
Gesture 38
+4 (Right Hand)
L0
NULL
R1
Gesture 39
+5 (Right Hand)
L0
NULL
R2
Gesture 40
+6 (Right Hand)
L0
NULL
R4
Gesture 41
+7 (Right Hand)
L0
NULL
R4
Gesture 42
+8 (Right Hand)
L0
NULL
R4
Gesture 43
+9 (Right Hand)
L0
NULL
R4
Gesture 44
-1 (Right Hand)
L0
NULL
R2
Gesture 45
-2 (Right Hand)
L0
NULL
R2
Gesture 46
-3 (Right Hand)
L0
NULL
R2
Gesture 47
-4 (Right Hand)
L0
NULL
R2
Gesture 48
-5 (Right Hand)
L0
NULL
R3

Gesture 49 -6 (Right Hand)

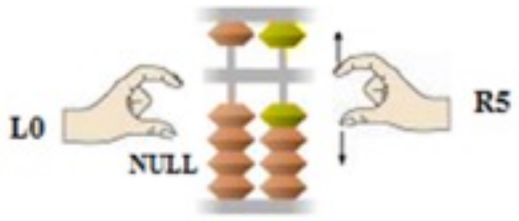

Gesture 50 -7 (Right Hand)

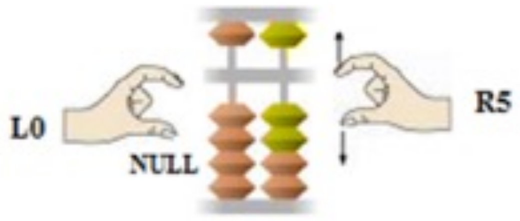

Gesture 51 -8 (Right Hand)

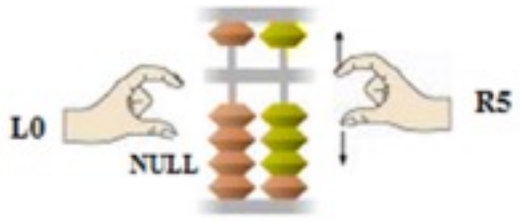

Gesture 52 -9 (Right Hand)

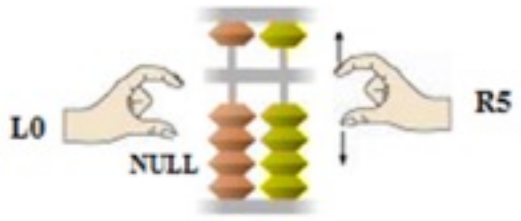

Gesture 53 +1 (Left Hand)

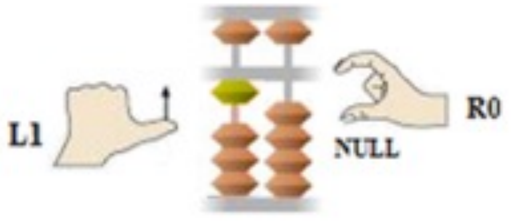

Gesture 54 +2 (Left Hand)

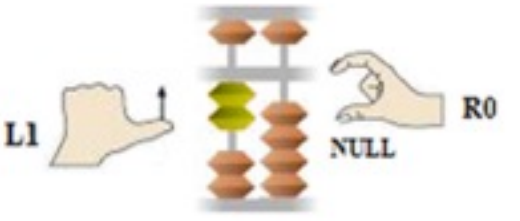

Gesture 55 +3 (Left Hand)

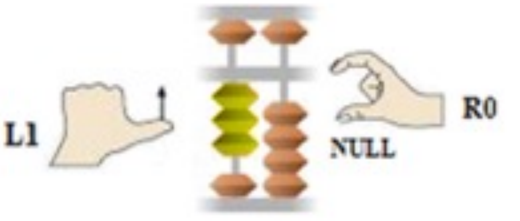

Gesture 56 +4 (Left Hand)

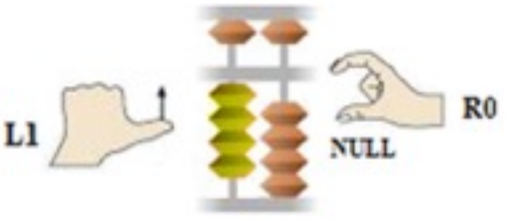

Gesture 57 +5 (Left Hand)

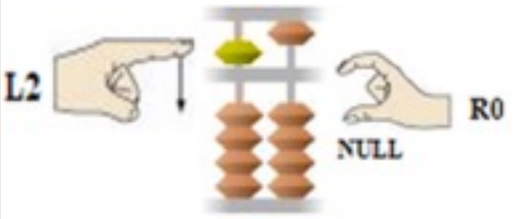

Gesture 58 +6 (Left Hand)

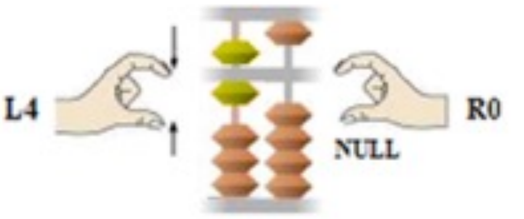

Gesture 59 +7 (Left Hand)

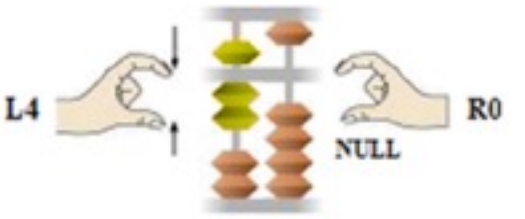

Gesture 60 +8 (Left Hand)

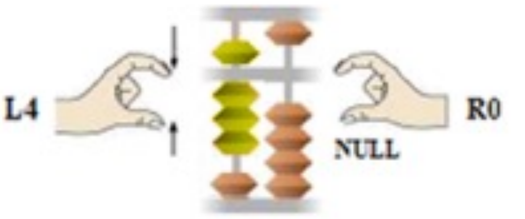

Gesture 61 +9 (Left Hand)

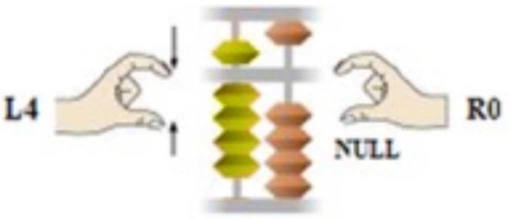

Gesture 62 -1 (Left Hand)

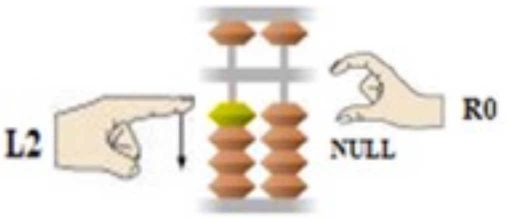

Gesture 63 -2 (Left Hand)

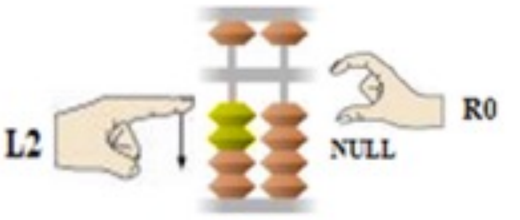

Gesture 64 -3 (Left Hand)

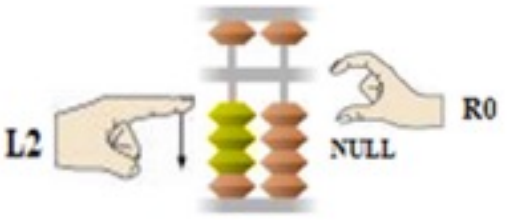

Gesture 65 -4 (Left Hand)

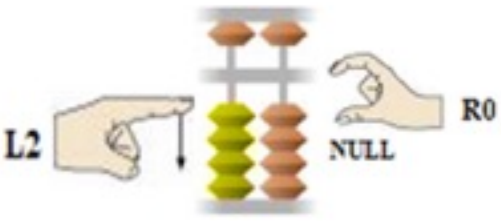

Gesture 66 -5 (Left Hand)

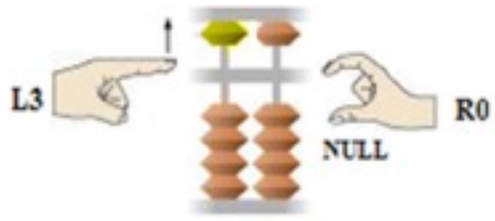

Gesture 67 -6 (Left Hand)

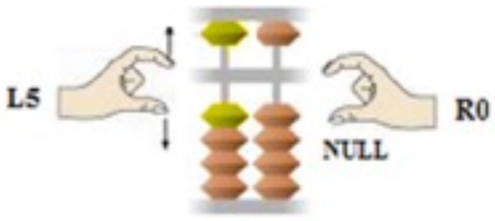

Gesture 68 -7 (Left Hand)

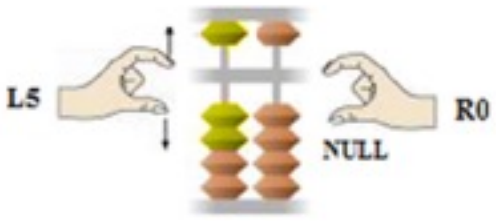

Gesture 69 -8 (Left Hand)

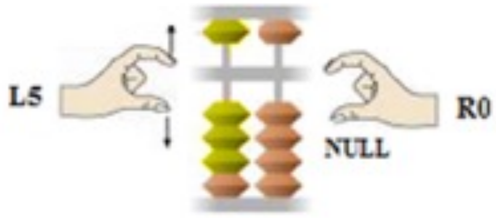

Gesture 70 -9 (Left Hand)

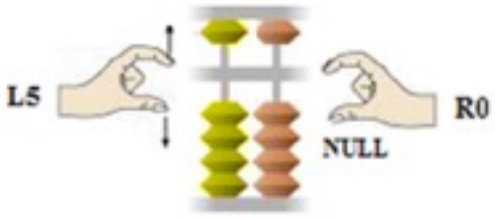

Supplemental Materials 2

Abacus Hand Movement Lexicon
&
Correct and Incorrect Gestures

Abacus Hand Movement Lexicon

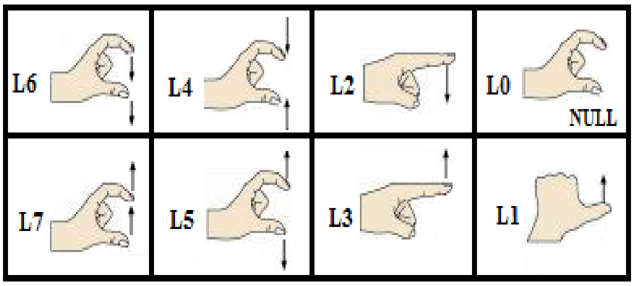

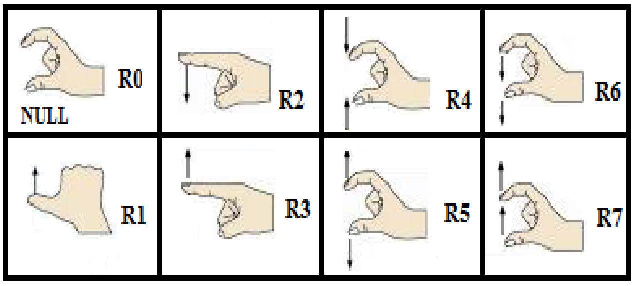

Two-handed combinations of these movements, using the index fingers and thumbs, form 70 abacus gestures (Supplemental Materials 1). Arrows show the direction of finger movements and colour indicates which beads have been moved. For example, R4 is a pinching motion. For R6, the index finger moves the upper-row bead down, while the thumb simultaneously moves lower-row beads down. Null means no movement.

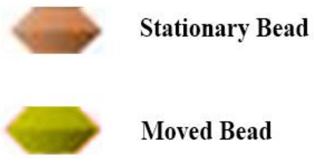

Example: Gesture 38 (Right Hand) +4

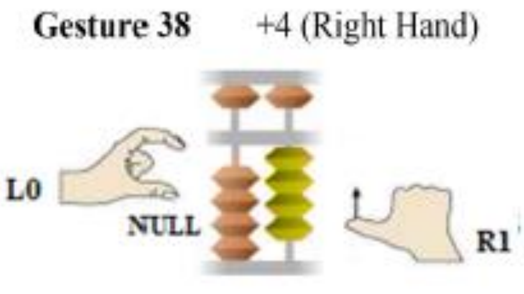

Correct

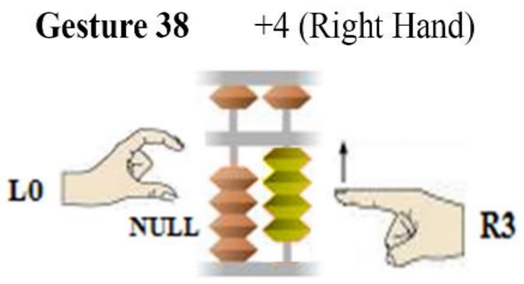

Incorrect

Note: The right thumb pushes 4 lower beads up. This is never done with the right index finger.

Example: Gesture 5 is +5= -5+10

(Note: This is used for a 5+5 operation. The complement +5-10 must be used.)

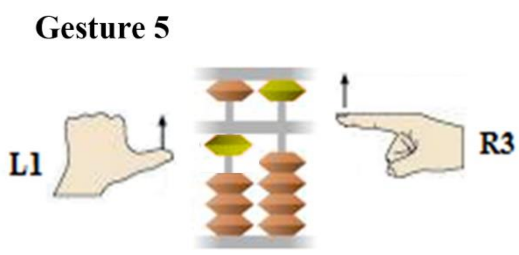

Correct

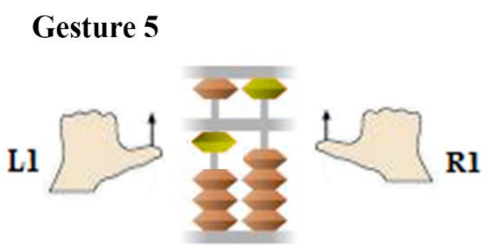

Incorrect

Note: The right index finger pushes the upper-row bead up. The right thumb does not do this.

Alternative Gestures

In each column, there is only one upper-row bead to register the value 5 and four lower-row of beads, each of which registers a digit-value of 1. If beads run out in a column, the value is carried over to the left or right adjacent column as a complement of 10. There are up to three different ways to add or subtract a digit value in a column. NOTE: ONLY ONE ALTERNATIVE GESTURE IS PHYSICALLY POSSIBLE, DEPENDING ON THE AVAILABILITY OF BEADS IN A COLUMN DURING A SPECIFIC STEP IN A CALCULATION.

Example: +1

There are three alternative gestures to add one in a column depending on the availability of beads.

Alternative One: +1 using Gesture 35.

This adds one directly with the right thumb when lower-row beads are available.

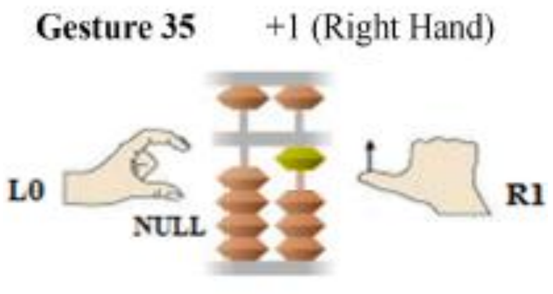

Alternative Two: +1 using Gesture 10

The calculation +4+1=5 can be performed as the gesture sequence 38, 10. Note that after performing Gesture 38, no beads are available to add one. So Gesture 10, the complement +5-4 must be used. After Gesture 10 is performed, the abacus will register 5.

4+1=5

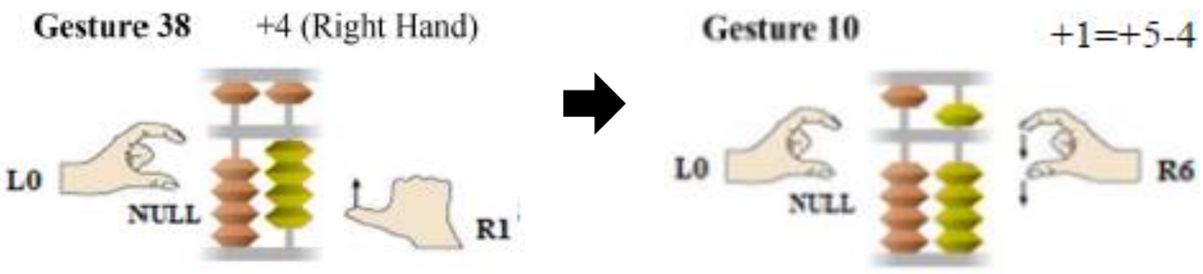

Step 1 **Step 2**

Alternative Three: +1 using Gesture 1

The calculation +9+1=10 can be performed as the gesture sequence 43, 1. Note that after performing Gesture 43, no beads are available to add one. So Gesture 1, the complement -9+10 must be used. After Gesture 1 is performed, the abacus will register 10.

9+1=10

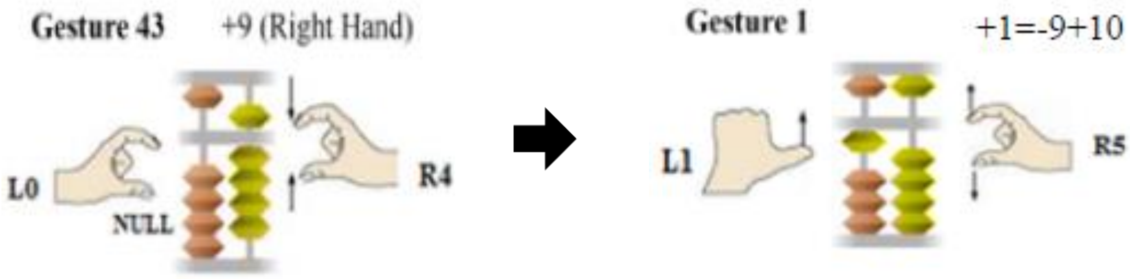

Step 1 **Step 2**

Note: In a two-handed method, the left hand can add a digit directly, when no complement is necessary. For example +10 can be performed as +1 with the left hand in the X10 column. This is Gesture 53. Likewise, +60 can be performed as +6 with the left hand, which is Gesture 58. The exponent depends on the column. The left and right hands operate on adjacent columns and move together from left to right.

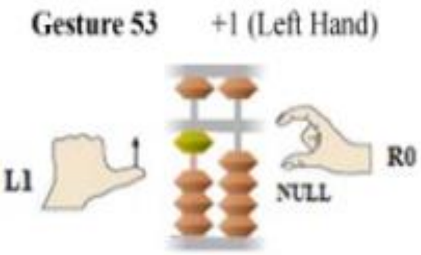

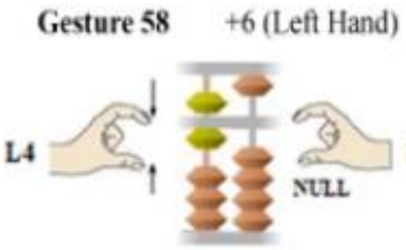

Supplemental Materials 3

How to Use an Abacus

&

Example Problems with Gesture Solutions

How to Use an Abacus

The *soroban* is the most popular contemporary form of abacus. Like a hand's five fingers, the abacus is composed of columns of five beads. The beads are separated into one upper row and four lower rows. In between the upper and lower rows is a horizontal bar, which is part of the rectangular frame. In the abacus picture below, this horizontal bar is labelled as the "value line". The array of beads is initialized to zero by setting the upper row against the upper part of the frame and the lower four rows against the bottom part of the frame. When a bead in the upper row is push downwards to touch the value line, it represents the digit-value of 5. When a bead in one of the four lower rows is pushed upwards towards the value line, each bead represents a digit-value of 1. A bead's value is subtracted when it is pushed away from the value line, back to the initial position. By adding the upper and lower beads together, each column can represent the digits 1 through 9. Starting with a one's column ($X10^0$), each column to the left is a successive power of ten (X10, X100, X1000, $X10^n$) and each column to the right a negative power of ten (X.1, X.01, X.001, $X10^{-n}$).

Calculations involve using algorithms for adding and subtracting complements of five and ten. These algorithms are taught as 70 two-handed gestures for simultaneously moving the beads in two adjacent columns at a time. These two-handed gestures use the index fingers and thumbs of both hands, in a combination of a few simple movements. There are up to 3 alternative algorithmic gestures to add a particular digit value, depending on the arrangement of beads prior to each operation. For example, 1+1 involves using the right thumb to push up one bead. This can be coded as Gesture 35 (see Supplemental Materials 1), written as movement (L0, R1) (see Supplemental Materials 2). However, to calculate 4+1, four lower-row beads must be first be pushed upwards with the right thumb using Gesture 38. Note that this leaves no available beads in the lower row to add 1. So the complement +5-4, must be used to add 1. This is represented as Gesture 10, movement (L0, R6), using the right index finger to push down the upper row bead (+5) and simultaneously using the right thumb to push down the four lower row beads to their original position (-4). An alternative algorithm to add the value 1 can be demonstrated by calculating 9+1. The beads to represent the value 9 must first be set by using gesture 43. This gesture uses the right index finger and thumb in a pinching movement (L0, R4) to push the upper bead and all four lower beads to the horizontal bar. Once the value 9 is set, however, there are no beads left in the column to add 1. So, the algorithm -9+10 must be used, pushing all the beads in the right hand column back to the original position (-9) and pushing one bead up in the left hand column (+10). This is performed by the two-handed gesture 1, which involves simultaneously moving the left thumb upwards and spreading the right-hand index finger and thumb away from each other, as a reverse pinch (L1, R5).

As Stigler has noted, any arithmetic calculation can then be performed as a fixed set of steps applying abacus algorithms. Each of these algorithmic steps can be coded as a sequence of gestures. For example, the four one-digit calculation 6+3+1+9 can be coded as the gesture sequence 40, 37, 1, 43. The four two-digit calculation 39+48+75+64 can be coded as gesture sequence 55, 43,13,8,7,5,14,13. Note that calculations proceed from left to right columns.

The *Soroban* Abacus

Columns multiply digit value by: X100 X10 X1 X.1

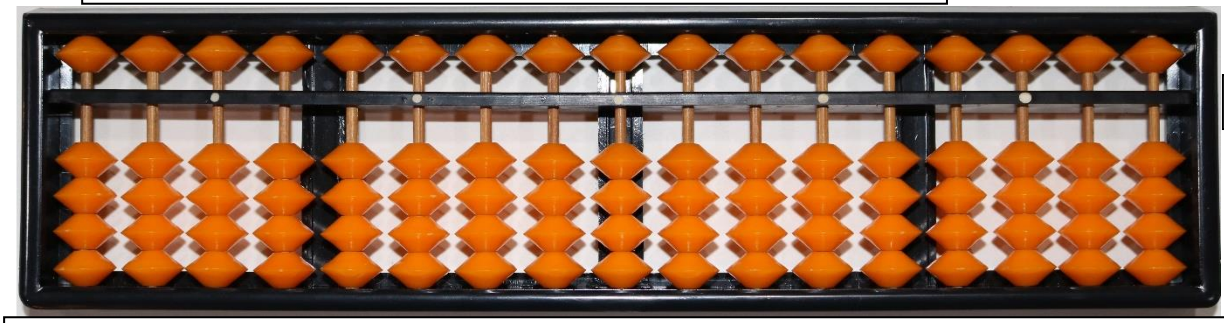

Upper row
Digit-value 5

Value Line

Lower row
Digit-value 1
each bead

Values are counted when an upper 5-bead is pushed down or a lower 1-bead is pushed up to touch the value line.

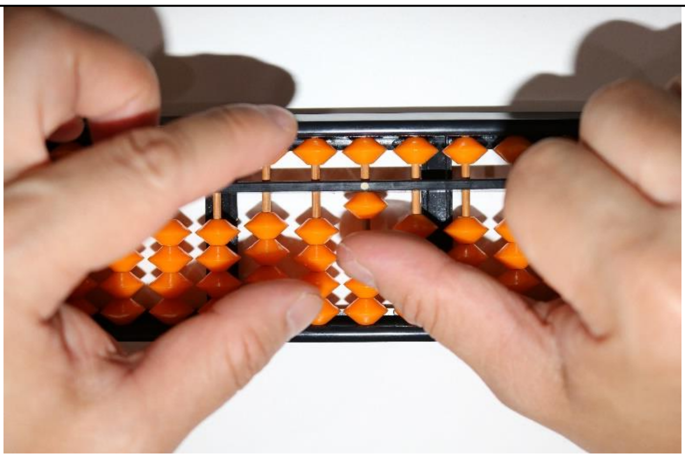

Value 1

Right thumb pushes a 1-bead up

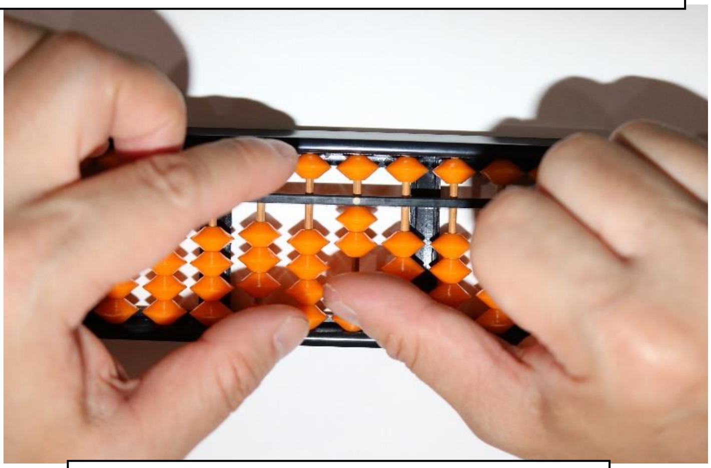

Value 2

Right thumb pushes two 1-beads up

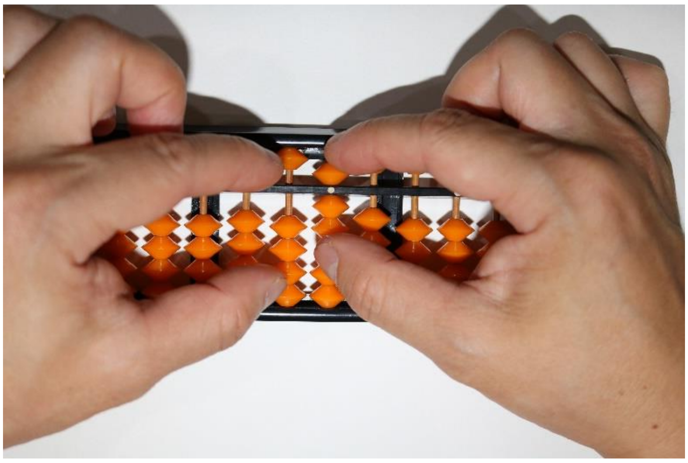

Value 7

Right index finger pushed one 5-bead down and two 1-beads up, in a pinching motion.

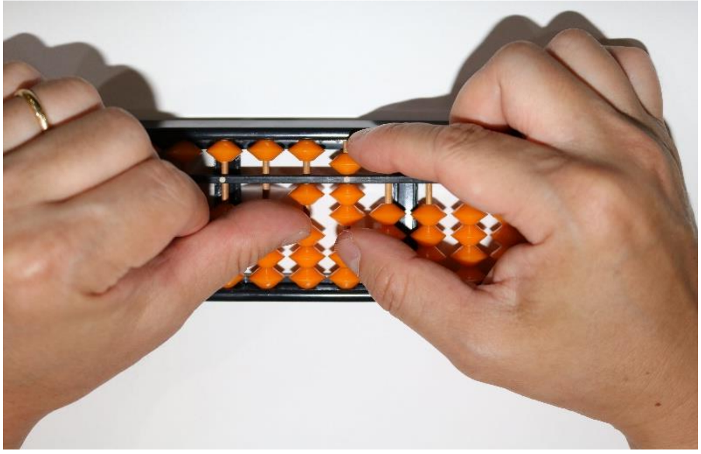

Value 17

Left thumb pushes 1-bead up in the X10 column. Right hand adds 7.

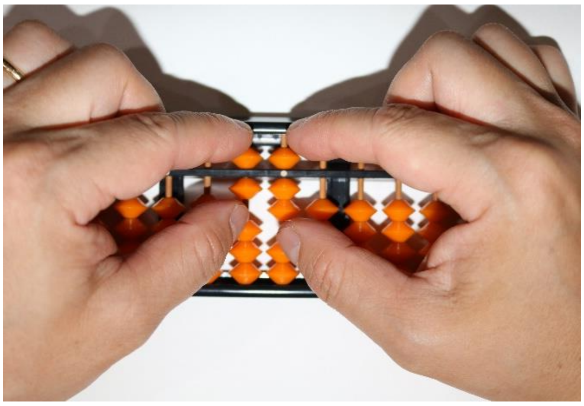

Value 67

Note the left hand over the X10 column uses the index finger to move a 5-bead down and thumb to move a 1-bead up, in a pinching motion. This is 50+10= 60. The right hand, likewise, pinches 7. Hence, the two columns register 67.

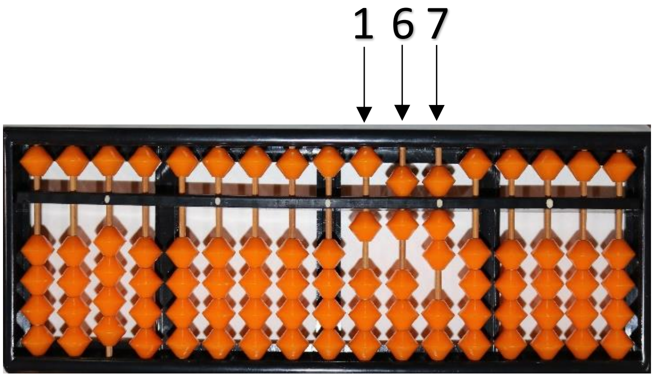

Value 167

Note that when the thumb in the X100 column pushes a 1-bead up, this registers 100.

Example 1-Digit Problem

Problem: 6+3+1+9 = 19

Gesture Sequence: 40, 37, 1, 43

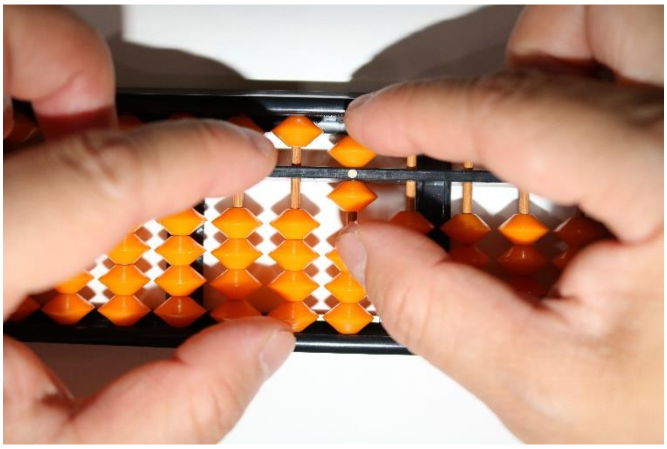

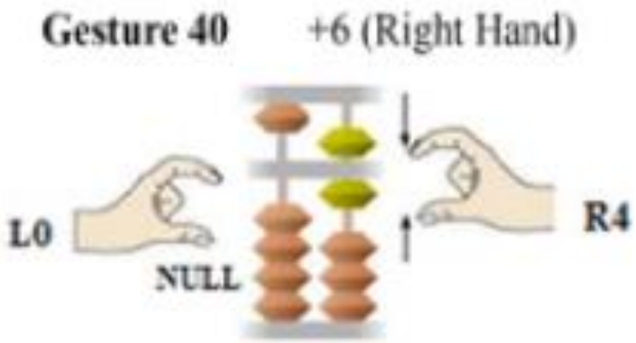

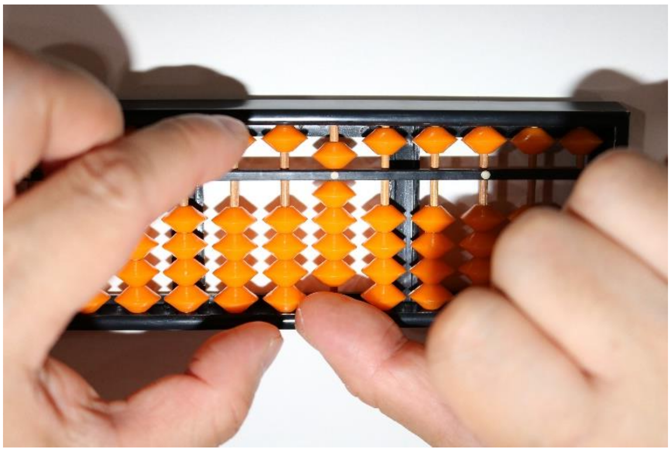

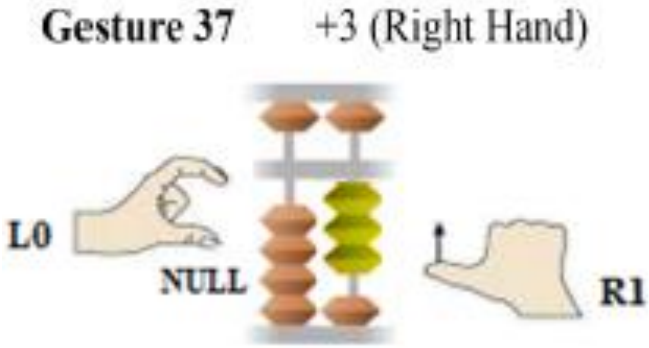

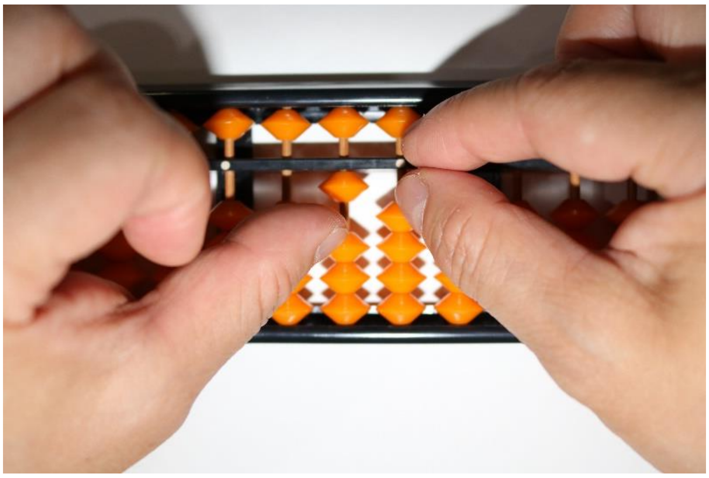

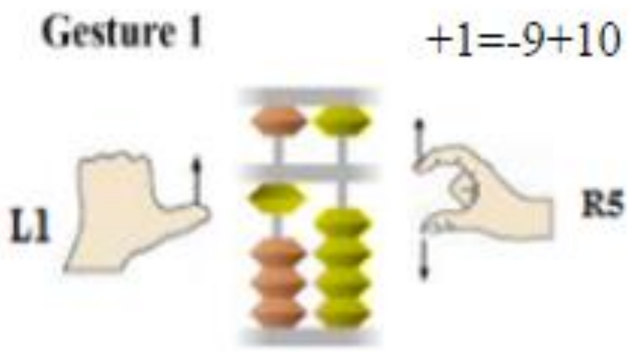

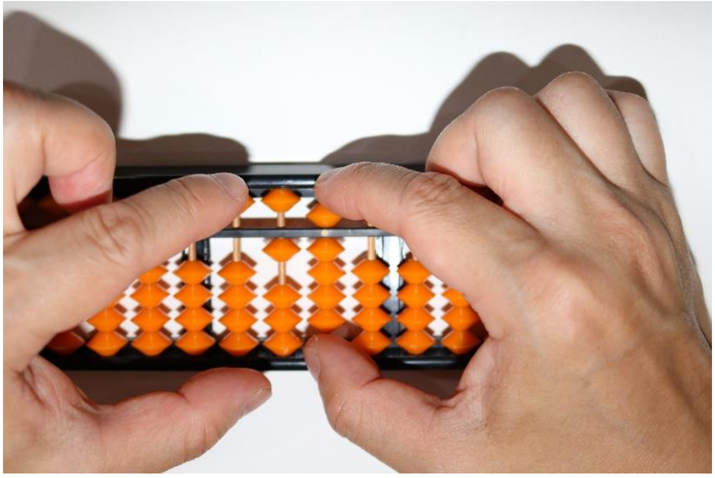

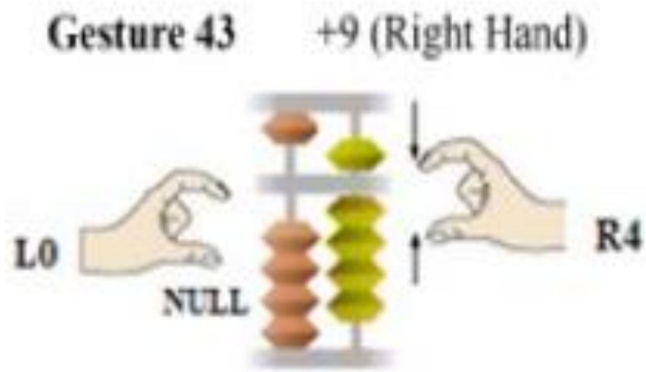

Note: Final value is 19

Example 2-Digit Problem

Problem: 39+48+75+64

Gesture Sequence: 55, 43,13,8,7,5,14,13

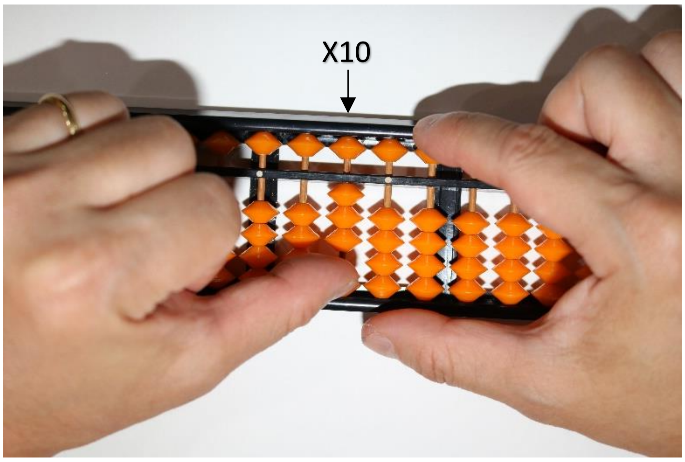

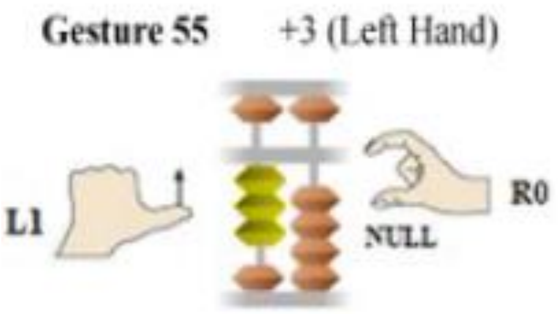

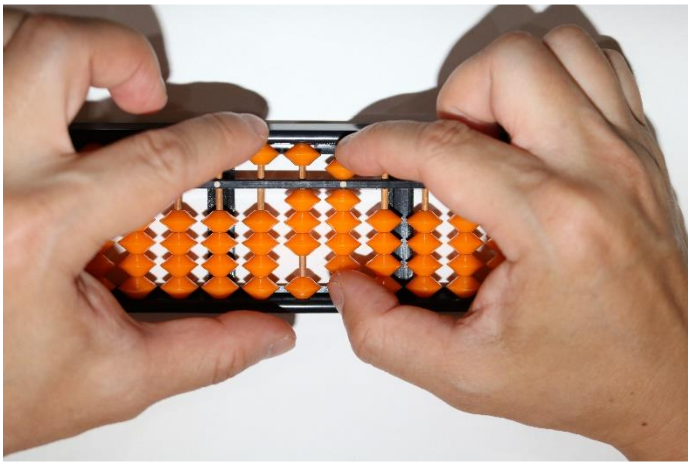

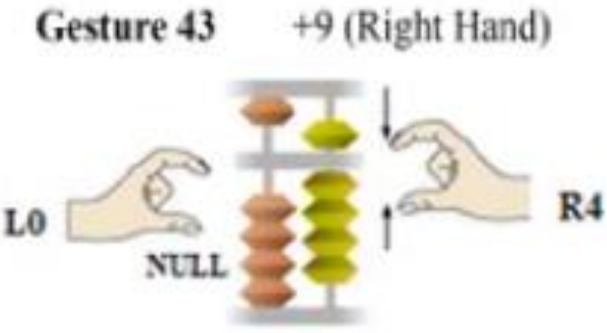

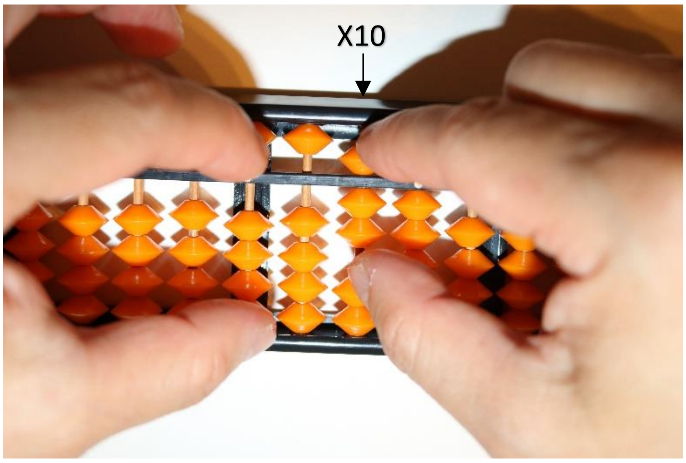

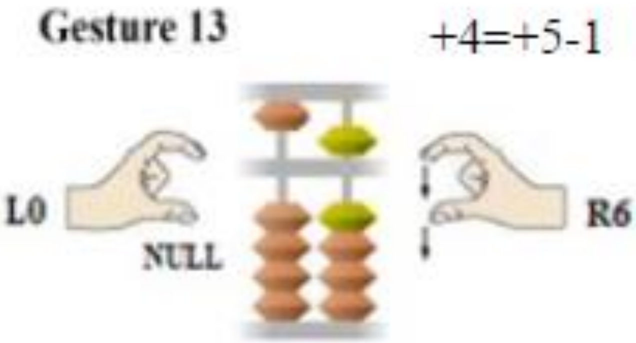

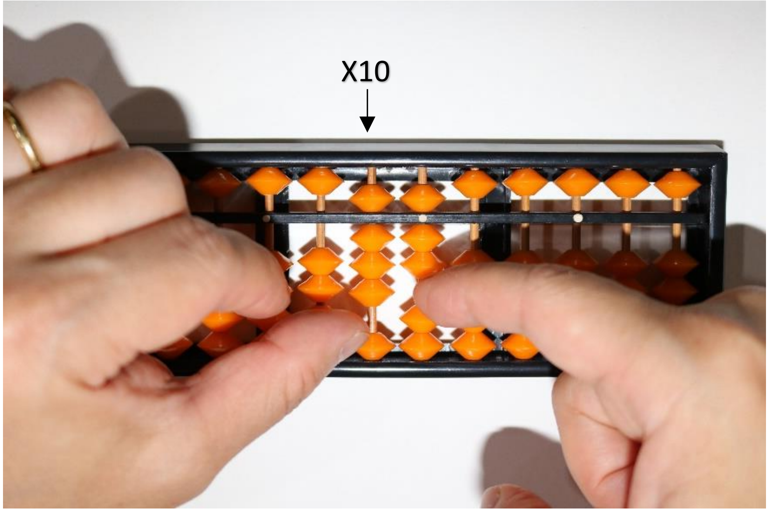
X10

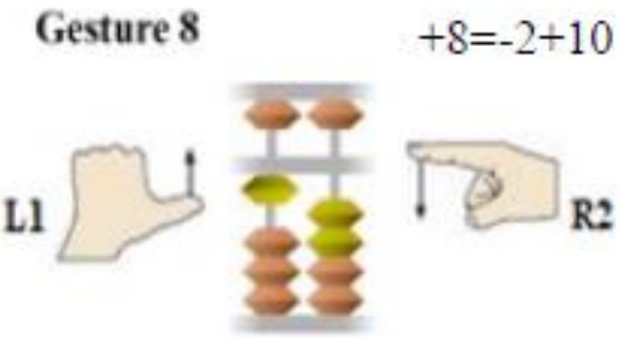
Gesture 8
+8=-2+10
L1
R2

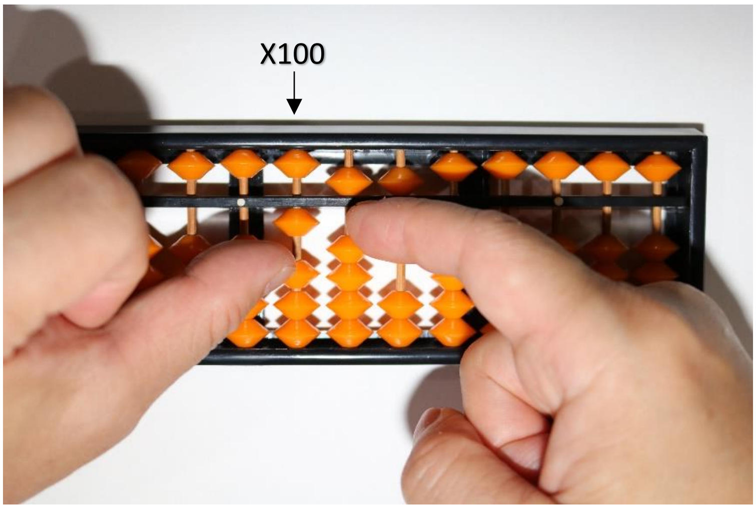
X100

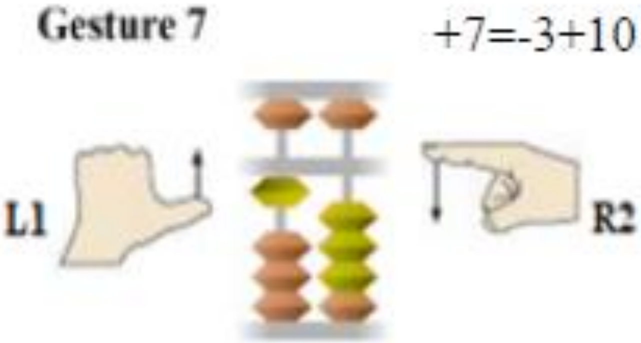
Gesture 7
+7=-3+10
L1
R2

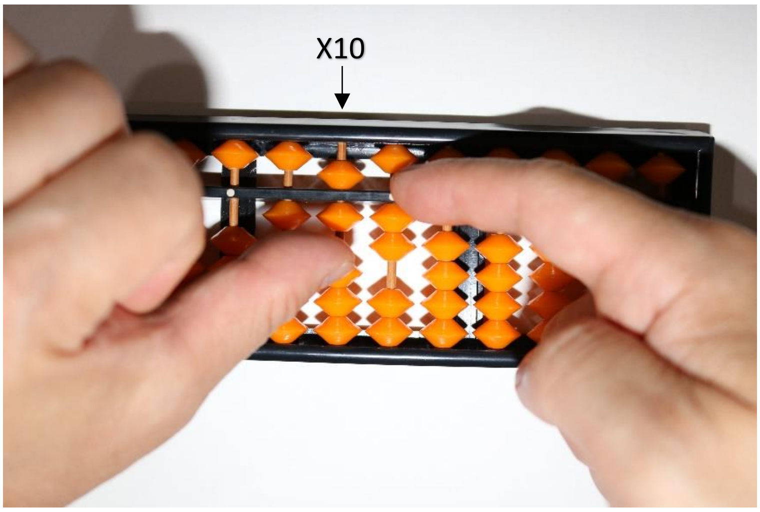
X10

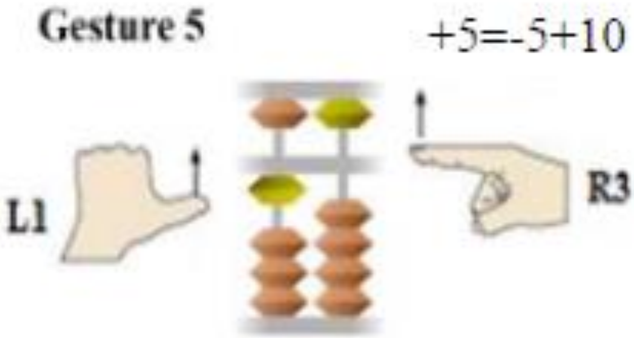
Gesture 5
+5=-5+10
L1
R3

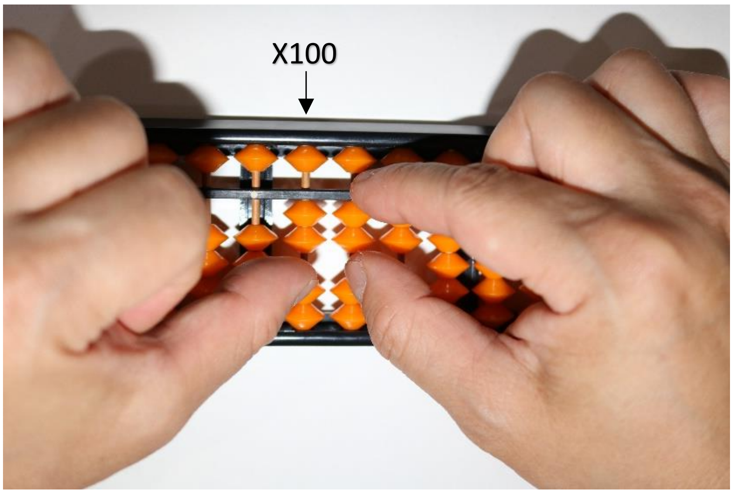

Gesture 14 +6=+1-5+10

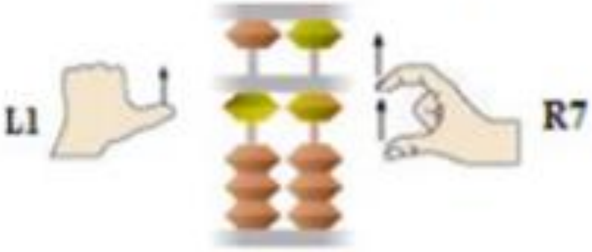

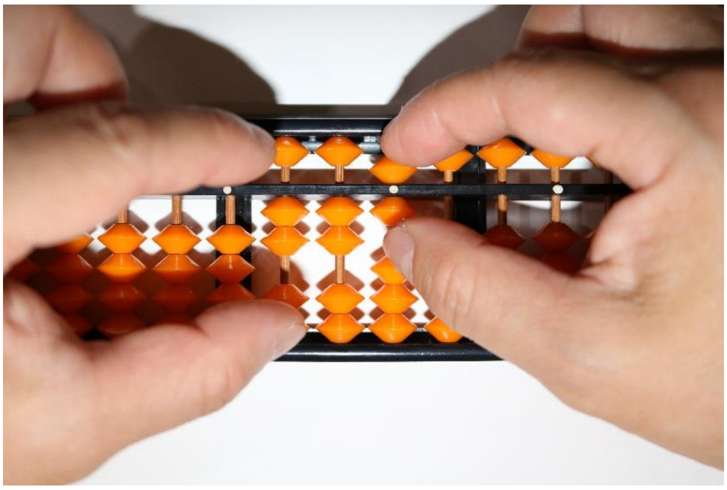

Gesture 13 +4=+5-1

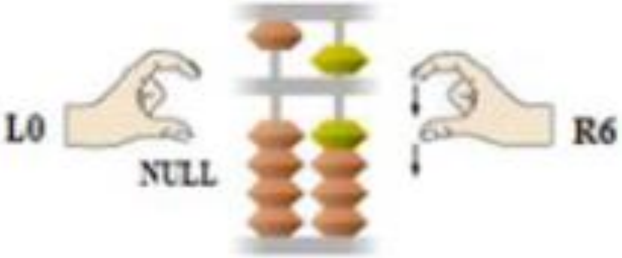

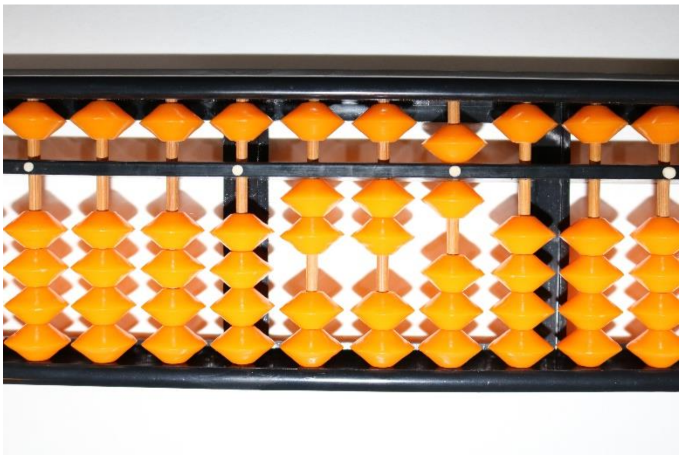

226

Historical Note

Embodied mathematical cognition as sociohistorical practice

The abacus is an anachronism that thrives in the 21st century because of technological choice and a tradition of innovation in embodied mathematics. One of a variety of hand-calculating methods in Asia, the abacus was originally a ritual object for fortune-tellers and philosophers to divine the heavens and fate. With the rise of 15th century inter-Asian commerce, however, the abacus became a popular tool for even merchants and shopkeepers, as evident in textbooks focusing on practical applications for trading. By the early 20th century, the various forms of abacus, such as the Japanese sorobon, were symbols of both national and, for the emerging field of accounting, professional identity. Accountants, who were mostly vocationally trained, were required to learn the abacus and continued to rely on it to double check transactions, even after the introduction of the electronic calculator. By the 1960s and 70s, western-educated mathematicians on national education boards in Asia began to phase out abacus training from school curriculums, sparking debates over modernization and cultural heritage. To survive, abacus teachers by the 1990s rebranded mental arithmetic as a form of early-childhood education and brain development. Independent *bushiban*, extra-curricular training centers, continue to market new hand techniques and patent different kinds of abacus to reinvent tradition. Through such historical contingencies, the abacus has become a cultural artifact that affects how the brain processes mathematical calculations as an embodied practice which continues to be socially reproduced in global networks of educational franchises.
